# Design, synthesis and evaluation of novel 2-phenyl-3-(1*H*-pyrazol-4-yl) pyridine positive allosteric modulators for the M_4_ mAChR

**DOI:** 10.1016/j.ejmech.2023.115588

**Published:** 2023-07-01

**Authors:** Manuela Jörg, Emma T. van der Westhuizen, Yao Lu, K.H. Christopher Choy, David M. Shackleford, Elham Khajehali, Andrew B. Tobin, David M. Thal, Ben Capuano, Arthur Christopoulos, Celine Valant, Peter J. Scammells

**Affiliations:** a Medicinal Chemistry, Monash Institute of Pharmaceutical Sciences, Monash University, Parkville, 3052, Victoria, Australia; b Drug Discovery Biology, Monash Institute of Pharmaceutical Sciences, Monash University, Parkville, 3052, Victoria, Australia; c ARC Industrial Transformation Training Centre for Cryo-Electron Microscopy of Membrane Proteins, Monash Institute of Pharmaceutical Sciences, Monash University, Parkville, 3052, Victoria, Australia; d Centre for Drug Candidate Optimisation, Monash Institute of Pharmaceutical Sciences, Monash University, Parkville, 3052, Victoria, Australia; e Centre for Translational Pharmacology, Institute of Molecular, Cell and Systems Biology, College of Medical, Veterinary and Life Sciences, University of Glasgow, Glasgow, G12 8QQ, United Kingdom; f Neuromedicines Discovery Centre, Monash Institute of Pharmaceutical Sciences, Monash University, Parkville, 3052, Victoria, Australia

**Keywords:** Acetylcholine, Allosteric ligands, M_4_ muscarinic acetylcholine receptor, Positive allosteric modulators, Xanomeline

## Abstract

Translation of muscarinic acetylcholine receptor (mAChR) agonists into clinically used therapeutic agents has been difficult due to their poor subtype selectivity. M_4_ mAChR subtype-selective positive allosteric modulators (PAMs) may provide better therapeutic outcomes, hence investigating their detailed pharmacological properties is crucial to advancing them into the clinic. Herein, we report the synthesis and comprehensive pharmacological evaluation of M_4_ mAChR PAMs structurally related to **1e**, Me–C-c, [^11^C]MK-6884 and [^18^F]12. Our results show that small structural changes to the PAMs can result in pronounced differences to baseline, potency (pEC_50_) and maximum effect (*E*_max_) measures in cAMP assays when compared to the endogenous ligand acetylcholine (ACh) without the addition of the PAMs. Eight selected PAMs were further assessed to determine their binding affinity and potential signalling bias profile between cAMP and *β*-arrestin 2 recruitment. These rigorous analyses resulted in the discovery of the novel PAMs, **6k** and **6l**, which exhibit improved allosteric properties compared to the lead compound, and probative *in vivo* exposure studies in mice confirmed that they maintain the ability to cross the blood-brain barrier, making them more suitable for future preclinical assessment.

## 1 Introduction

Centrally expressed muscarinic acetylcholine receptors (mAChRs) have emerged as novel drug targets for neurodevelopmental and neurodegenerative diseases such as schizophrenia and Alzheimer’s disease, respectively. mAChRs are members of the G protein-coupled receptor (GPCR) family of proteins that respond to the neurotransmitter, acetylcholine (ACh). There are five subtypes of mAChRs (M_1_-M_5_), which preferentially couple to either G_*αq*_ (M_1_, M_3_ and M_5_ mAChRs) or G_*αi/o*_ (M_2_ and M_4_ mAChRs) proteins to initiate signalling on downstream pathways, although can also display additional promiscuous signalling properties. mAChRs are ubiquitously expressed, with the M_1_, M_3_, M_4_ and M_5_ subtypes most abundant in the CNS, while M_2_ and M_3_ are more relevant for cholinergic function in the periphery [1,2].

In human clinical trials, the ‘M_1_/M_4_ mAChR-preferring’ agonist, xanomeline, reduced psychosis, behavioural disturbances and improved cognitive function in patients with either Alzheimer’s disease or schizophrenia [3,4]; supporting the hypothesis that mAChRs are promising targets for the development of novel drug candidates. Unfortunately, xanomeline failed initial Alzheimer’s disease clinical trials due to intolerable cardiovascular and gastrointestinal adverse effects; although xanomeline is an M_1_/M_4_-preferring ligand, it also binds with equal affinity to the orthosteric site of other mAChR subtypes, albeit with lower efficacy [5]. Recently a new combination therapy, consisting of xanomeline plus a peripherally restricted orthosteric mAChR antagonist, trospium (KarXT), sought to overcome this selectivity-based limitation and indeed passed phase II human clinical trials for the treatment of schizophrenia [6]. KarXT improved cognition and psychosis in schizophrenic patients through the action of xanomeline, whilst trospium reduced the peripheral adverse effects of xanomeline on peripherally expressed mAChRs [6].

An alternative to overcoming the limitations associated with the high conservation of the orthosteric site across the five mAChR subtypes is the pursuit of more selective compounds that bind to spatially distinct allosteric sites. These sites do not have the same degree of amino acid residue conservation as the orthosteric site, allowing for improved potential in subtype-selective ligand development [7,8]. Because allosteric ligands can bind to the mAChRs concomitantly with an orthosteric ligand, such as ACh, they can differentially modulate affinity and/or efficacy of the co-bound orthosteric ligand [9]. Allosteric modulators that increase the affinity and/or efficacy of orthosteric ligands are termed positive allosteric modulators (PAMs), allosteric modulators that decrease orthosteric ligand affinity and/or efficacy are termed negative allosteric modulators (NAMs), while allosteric ligands that bind to the allosteric site but do not modulate orthosteric ligand affinity/efficacy are termed neutral allosteric ligands (NAMs) [10].

**Fig. 1:**
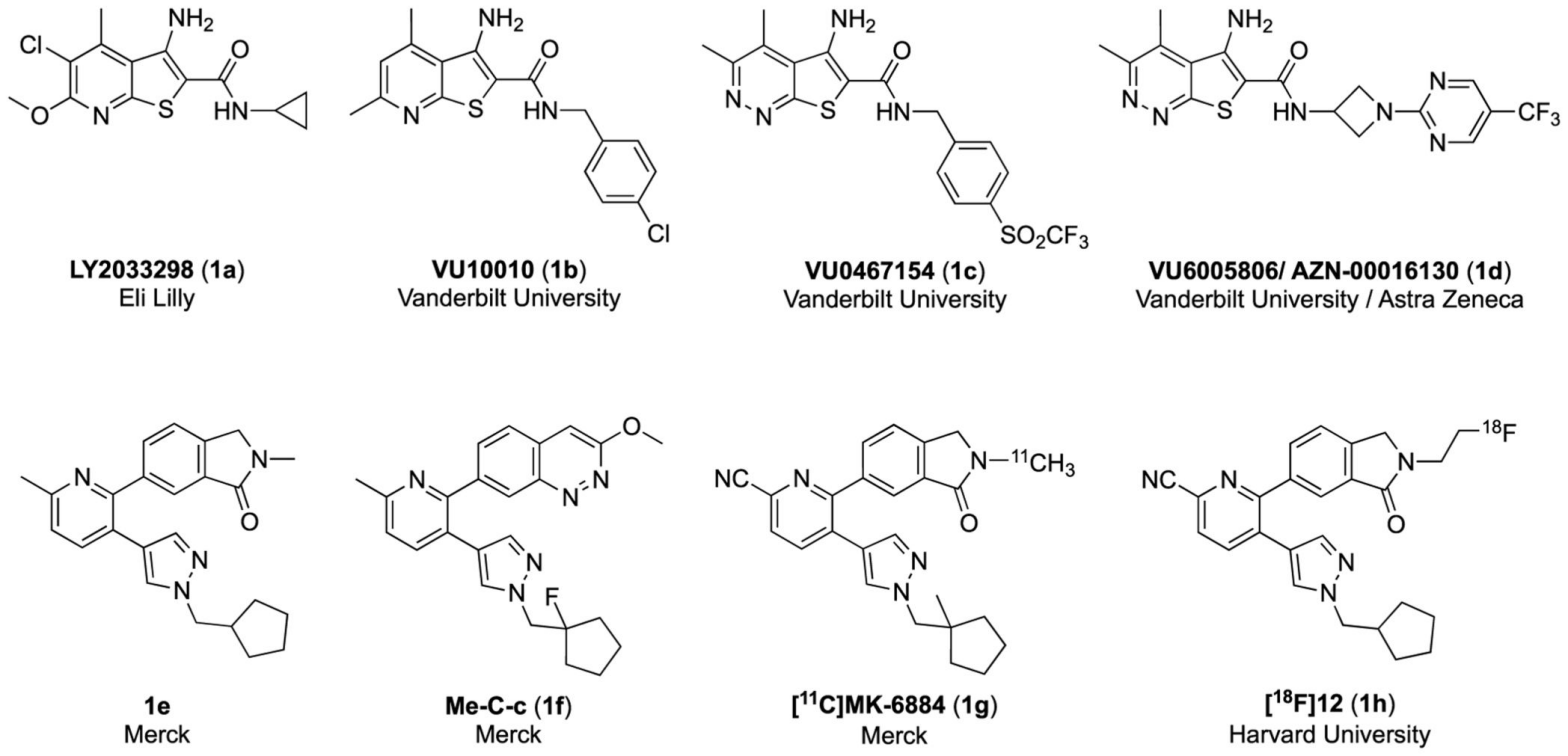
Chemical structures of published M_4_ mAChR PAMs. LY2033298 by Eli Lily, VU10010, VU6005806, VU0467154 by Vanderbilt University and compound **1e**, Me–C-c and [^11^C]-MK-6884 by Merck and [^18^F]12 by researchers at Harvard University.

The first very highly selective PAM for the mAChRs was the M_4_ mAChR preferring PAM, LY2033298, developed by Eli Lilly (Fig. 1), which also had efficacy in preclinical models commonly used to evaluate schizophrenia symptom domains [11]. Unfortunately, further characterisation of LY2033298 revealed significant liabilities, such as poor brain penetration, intractable chemical scaffold and species variability, with LY2033298 modulating the effects of ACh at the human receptor to a greater extent than at the mouse M_4_ mAChR [12,13]. To improve the drug-like properties of LY2033298, the structurally related thieno[2, 3-*b*]pyridine (ML253) [14] and thieno[2,3-*c*]pyridazine-based (VU0467154) [15] (Fig. 1) M_4_ mAChR PAMs were developed by Vanderbilt University. However, while VU0467154 was promising in mouse behavioural studies, it again displayed significant species variability in its allosteric effects, preventing it from progressing beyond pre-clinical models [16]. Further work around this scaffold, yielded VU6005806/AZN-00016130, which like VU0467154 showed promise in rodent models, but had poor bioavailability in dogs and macaques, thus was discontinued as a candidate for human clinical trials [17]. These studies validate the M_4_ mAChR as a viable target for improving psychotic and cognitive symptoms in preclinical models, however, highlight the ongoing issues with species variability, bioavailability and pharmacokinetics that have hindered progression of M_4_ mAChR PAMs into the clinic.

Structural studies have been conducted to explore M_4_ mAChR activation by agonists, allosteric agonists and allosteric modulators [18]. More specifically, cryo-electron microscopy structures of the agonist iperoxo, a selective allosteric agonist and a positive allosteric modulator (LY2119620) bound to a M_4_ mAChR-G_i_ complex have revealed the binding modes of these ligands and provided insights into their mechanism of receptor activation.

A high throughput screening campaign identified (4-(3-(1-isopentyl-1*H*-pyrazol-4-yl)pyridin-2-yl)phenyl)methanamine as a possible candidate for further development [19–21]. Work around this scaffold by Merck resulted in the development of the tool compound, Me–C-c (Fig. 1), which exhibited good selectivity and potency for the human and rat M_4_ mAChRs in calcium mobilization assays conducted in the presence of an EC_50_ concentration of ACh. Furthermore, this compound was not a substrate of P-glycoprotein (P-gp) efflux [21]. Although the compound had good *in vivo* permeability, the bioavailability in rats was poor, resulting in the necessity of alternative delivery strategies to achieve high exposure in the brain. Further structural improvements allowed the development of a PET tracer, [^11^C]MK-6884, with good brain uptake, which was used in *in vivo* rhesus monkey studies to determine the plasma concentration and receptor occupancy [22]. More recently, an attempt to develop a clinical M_4_ mAChR PET tracer led to the development of the radiofluorinated probe [^18^F]12 [23]. While the probe showed good subtype selectivity for the M_4_ mAChR in *in vitro* autoradiography studies, species variability was nonetheless observed in the presence of the orthosteric agonist, carbachol. These studies exemplify the importance of the detailed characterisation PAMs to advance the development of both diagnostic probes and therapeutic candidates targeting the M_4_mAChR.

**Fig. 2:**
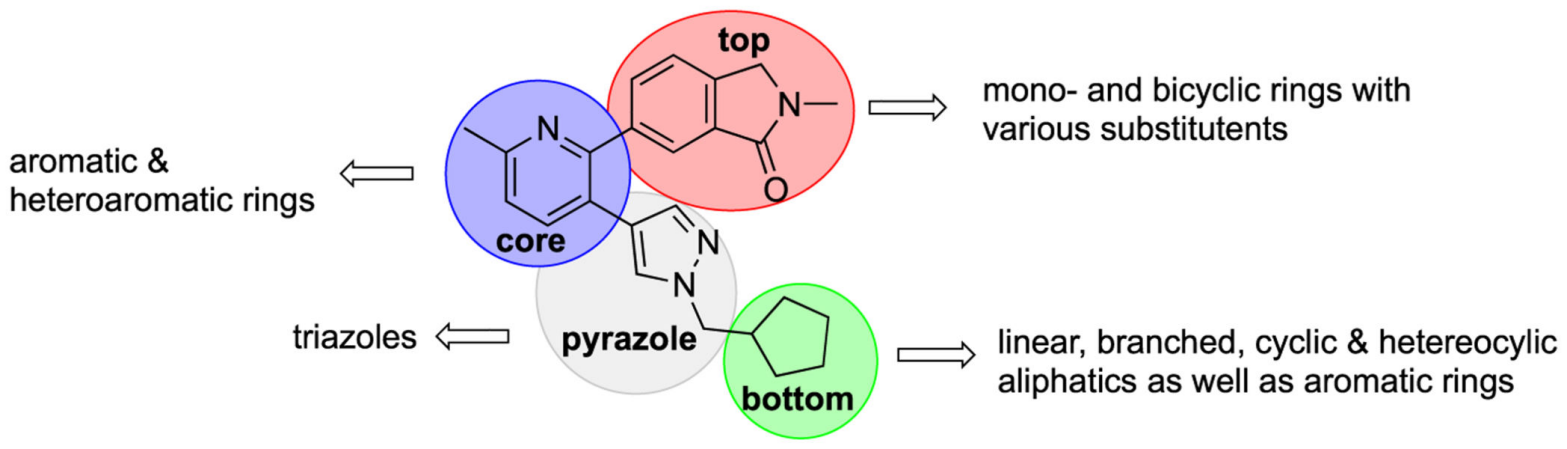
Overview of the structural modifications to the lead compound **1e**.

Herein, we present the detailed evaluation of the structure-activity relationship data of M_4_ mAChR PAMs based on the common 2-phenyl-3-(1*H*-pyrazol-4-yl)pyridine scaffold of **1e**, Me–C-c, [^11^C]MK-6884 and [^18^F]12. Investigated were structural modifications to the top, core, or bottom regions as well as the pyrazole ring of our lead (Fig. 2). Key modifications that have not been explored previously, included alterations to 2-methylisoindolin-1-one motif, the replacement of the pyridine core pendant with other six-membered aromatic heterocycles, and substitution of the pyrazole moiety with a triazole ring. The synthesised analogues were not only assessed for their affinity at the M_4_ mAChR, but also their unique allosteric profile, consisting of the degree of allosteric agonism (change in baseline), and their ability to potentiate the potency and maximal effect of ACh. Furthermore, signalling bias profiles in two functional assays, cAMP signalling and *β*-arrestin 2 recruitment, were assessed for selected compounds. Finally, selected compounds were progressed to *in vivo* exposure studies.

## 2 Results and discussion

### 2.1 Chemistry

**Scheme 1:**
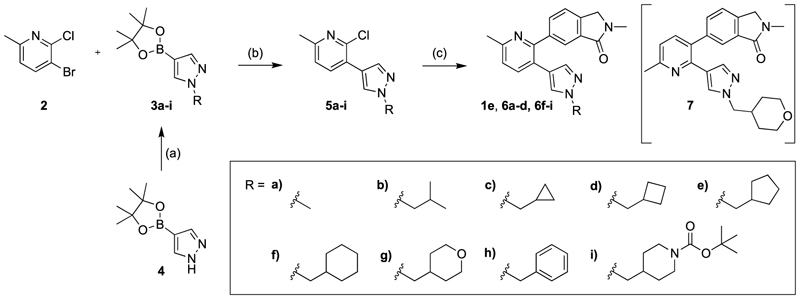
Synthesis of analogues with different bottom motifs. Reagents and conditions: (a) respective halide, K_2_CO_3_, DMF, 60 °C, 32–68%; (b) respective pinacol ester, cat. PdCl_2_(PPh_3_)_2_, Cs_2_CO_3_, DMF or DME, 100 °C, 12–46%; (c) 2-methyl-6-(4,4,5,5-tetramethyl-1,3,2-dioxaborolan-2-yl)isoindolin-1-one, cat. PdCl_2_(PPh_3_)_2_, Cs_2_CO_3_, DMF or DME, 100 °C, 4–82% (for **6g**, compound **7** was isolated as a by-product, which resulted from the presence of a minor amount of the unwanted regioisomer in the precursor, **5g**).

**Scheme 2:**
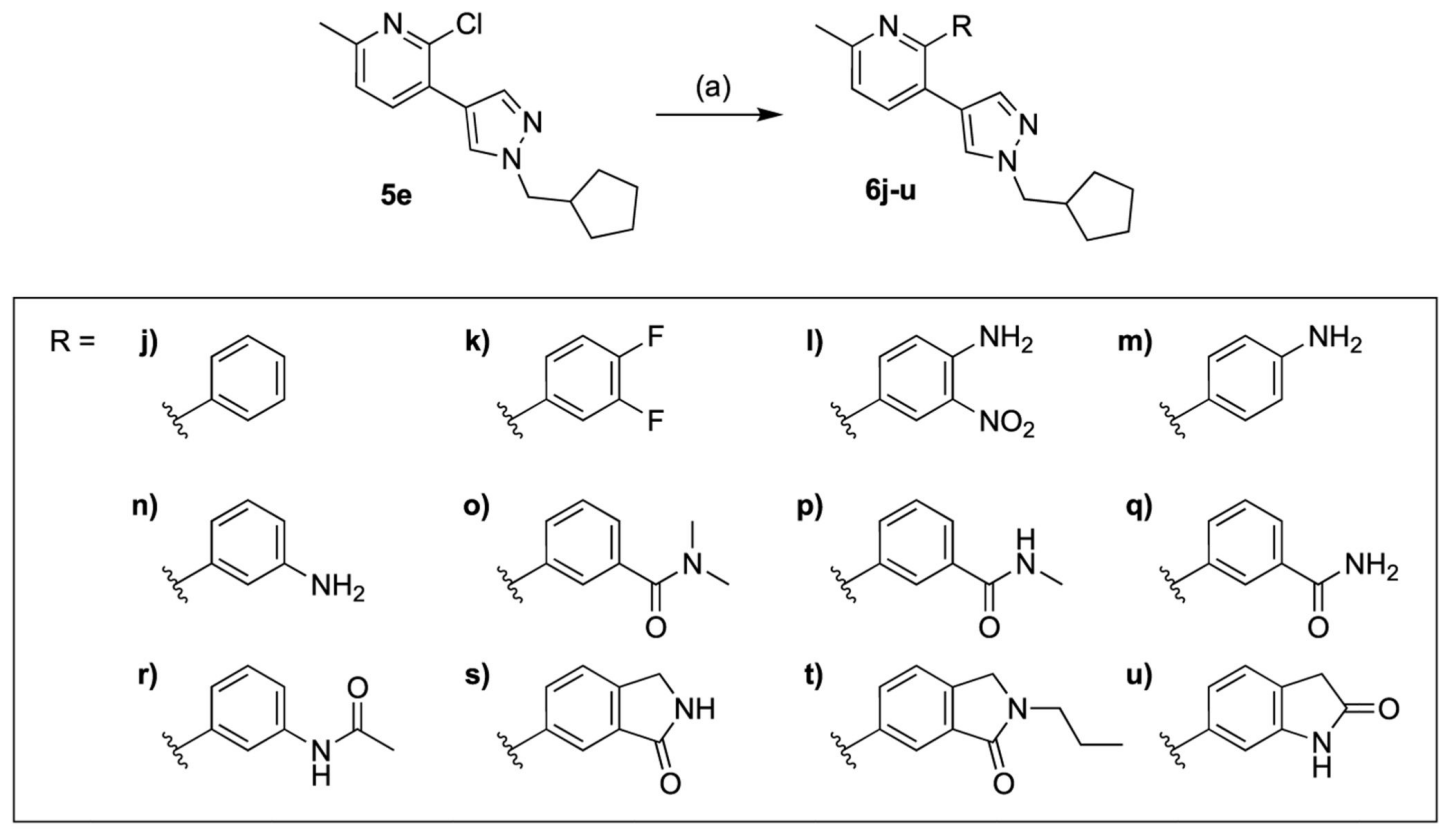
Synthesis of analogues with different top motifs. Reagents and conditions: (a) respective boronic acid or pinacol ester, cat. PdCl_2_(PPh_3_)_2_, Cs_2_CO_3_, DMF or DME, 100 °C, 5–82%.

**Scheme 3:**
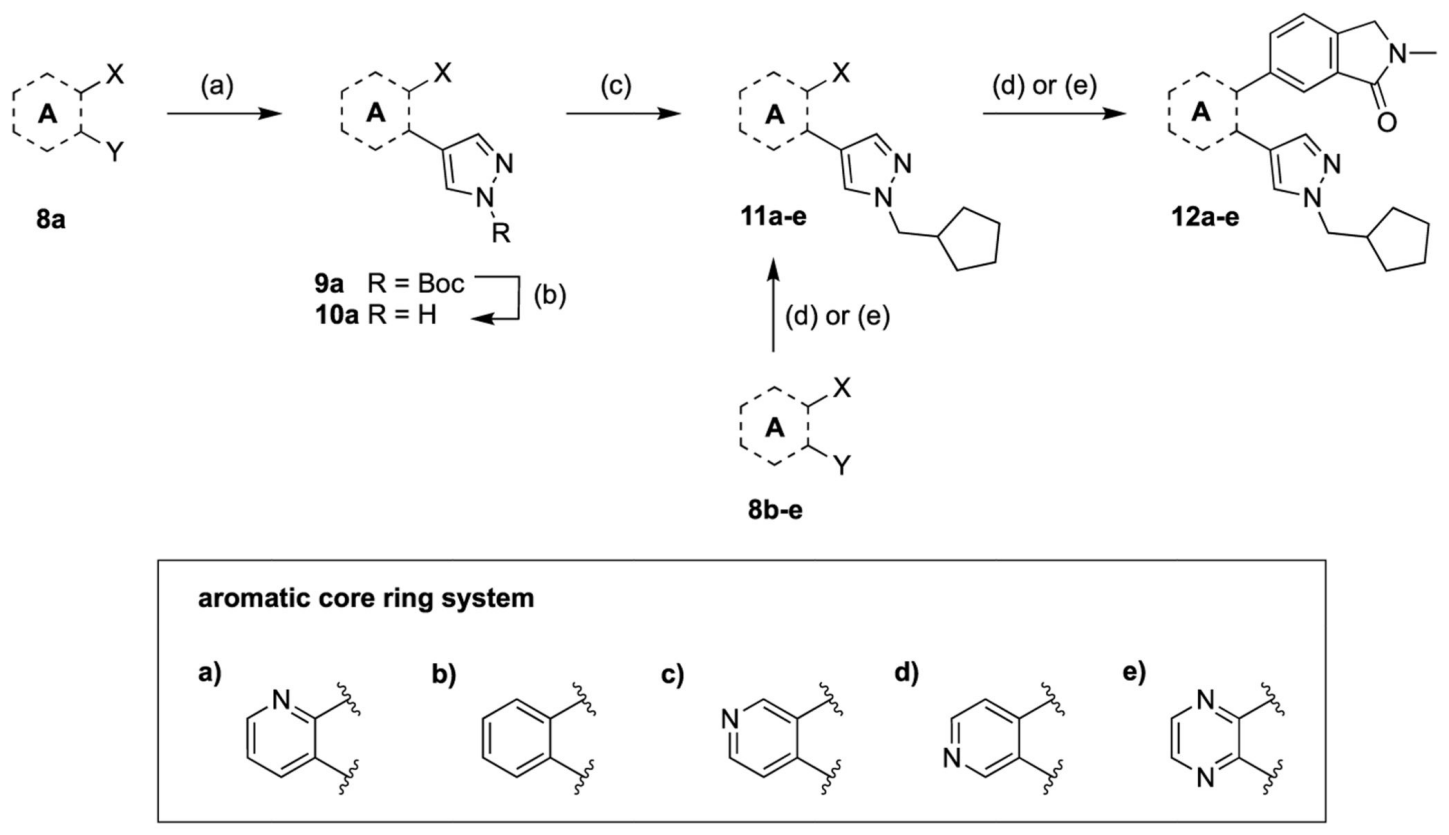
Synthesis of analogues with different core motifs. Reagents and conditions: (a) 1-Boc-pyrazole-4-boronic acid pinacol ester, cat. PdCl_2_(PPh_3_)_2_, 1 M Na_2_CO_3(aq)_/THF, 100 °C, 28%; (b) TFA, DCM, rt, 46%; (c) (bromomethyl)cyclopentane, K_2_CO_3_, DMF, rt-60 °C, 55%; (d) pinacol ester, cat. PdCl_2_(PPh_3_)_2_, Cs_2_CO_3_, DMF or DME, 100 °C, 16–63%; (e) pinacol ester, cat. PdCl_2_(PPh_3_)_2_, 1 M Na_2_CO_3(aq)_/THF, 100 °C, 7–8%.

The general synthetic pathway of the novel M_4_ mAChR allosteric ligands was based on two Suzuki coupling reactions to functionalise a range of *ortho*-dihalogenated aromatic rings (Schemes 1–3). First, the bottom pendant was installed using different pyrazole boronic acid pinacol esters, following the introduction of the top motif via a second Suzuki coupling reaction. We mostly used water-free reaction conditions for the Suzuki couplings to avoid the 2-chloropyridine precursors undergoing any potential nucleophilic aromatic substitution side-reactions promoted by the basic conditions. Alkylation of the pyrazole moiety was achieved both with the boronic ester **4** (Scheme 1) as well as intermediates **10a** (Scheme 3) resulting in respectable yields ranging between 32 and 68%. However, we generally alkylated the pyrazole boronic ester due to the ease of purification. Using this overall approach, we synthesised analogues with alterations to the bottom (Scheme 1), top (Scheme 2) and core motif (Scheme 3).

**Scheme 4:**
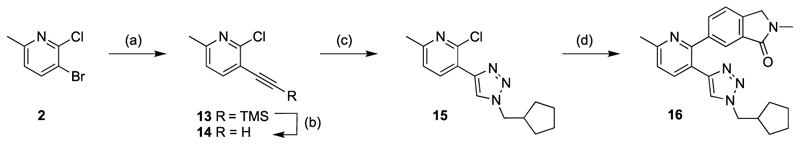
Synthesis of triazole analogue **16**. Reagents and conditions: (a) ethynyltrimethylsilane, Pd(PPh_3_)_2_Cl_2_, CuI, DIPEA, 100 °C, 30%; (b) K_2_CO_3_, MeOH, rt, 94%; (c) (azidomethyl)cyclopentane (1 M solution in 2-methoxy-2-methylpropane), CuSO_4_•5H_2_O, sodium ascorbate, *t*-BuOH:H_2_O 1:1, rt, 91%; (d) 2-methyl-6-(4,4,5,5-tetramethyl-1,3,2-dioxaborolan-2-yl)isoindolin-1-one, cat. PdCl_2_(PPh_3_)_2_, Cs_2_CO_3_, DMF, 100 °C, 33%.

Furthermore, we synthesised two analogues where the pyrazole moiety was replaced with a triazole ring. The triazole **16** (Scheme 4) was synthesised from 3-bromo-2-chloro-6-methylpyridine (**2**) using Sonogashira coupling reaction conditions to introduce TMS-acetylene. Neat reaction conditions were used to improve the conversion of the starting material **2** to the desired Sonogashira product **13**. Nevertheless, only a moderate yield of 30% was achieved due to the incomplete reaction conversion of the starting material, in addition to the formation of the undesired side product 3-bromo-6-methyl-2-((trimethylsilyl)ethynyl) pyridine, which made the purification via column chromatography challenging. Next, the TMS protecting group was removed under alkaline conditions to afford **14** in 94% yield. Click chemistry was used to form the triazole ring from intermediate **14** and (azidomethyl)cyclopentane in 91% yield. Lastly, the methyl-1-isoindolinone top motif was introduced via standard Suzuki reaction conditions in moderate yield (33%).

**Scheme 5:**
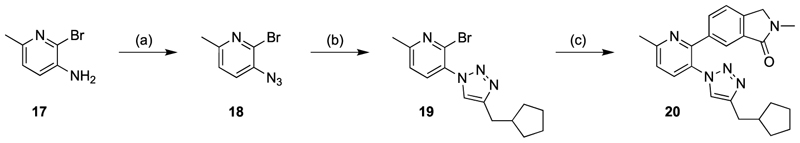
Synthesis of triazole analogue **20**. Reagents and conditions: (a) *tert*-butylnitrite, MeCN at 0 °C, followed by TMSN_3_, 0 °C – rt, quantitative yield; (b) prop-2-yn-1-ylcyclopentane, CuSO_4_•5H_2_O, sodium ascorbate, *t*-BuOH:H_2_O 1:1, rt, 27%; (c) 2-methyl-6-(4,4,5,5-tetramethyl-1,3,2-dioxaborolan-2-yl)isoindolin-1-one, cat. PdCl_2_(PPh_3_)_2_, Cs_2_CO_3_, DMF, 100 °C, 27%.

Triazole analogue **20** (Scheme 5) was synthesised starting from 2-bromo-6-methylpyridin-3-amine (**17**). Firstly, the amino functionality was converted to an azide using *tert*-butyl nitrite and azido-trimethylsilane to afford **18** in quantitative yield. Next, the triazole ring was formed by reacting azide **18** with prop-2-yn-1-ylcyclopentane to obtain intermediate **19**, which underwent a Suzuki coupling reaction with 2-methyl-6-(4,4,5,5-tetramethyl-1,3,2-dioxaborolan-2-yl)isoindolin-1-one to afford the final analogue **20** in 27% yield.

### 2.2 Pharmacology

#### 2.2.1 Two-concentration screening of the M_4_ PAMs

The new compounds were initially screened in Chinese-hamster ovary (CHO) cells stably expressing the human M_4_ mAChR. Activation of CAMYEL (cAMP sensor using YFP-Epac-RLuc) was selected as the signalling pathway for the initial screening because it is a weakly coupled pathway with low signal amplification at the hM_4_ mAChR [24–26]. In this system, ACh is a partial agonist relative to the high efficacy agonist, iperoxo (Supp. Fig. 1), thus allosteric effects can be observed at the level of direct efficacy from the modulator (changes in baseline), binding cooperativity (changes in potency, pEC_50_) and efficacy cooperativity (changes in *E*_max_). Initial allosteric modulator screening examined the effect of two concentrations (1 μM and 10 μM) of each modulator on the full ACh concentration-response curve (Supp. Figs. 2–4). The effects of the novel allosteric modulators were quantified as a change in baseline, ACh-mediated pEC_50_ and ACh maximal response, *E*_max_, which are shown in Tables 1–3.

Table 1 shows the changes in baseline, pEC_50_ and *E*_max_ for the compounds with alterations around the bottom pendant. Two distinct profiles of modulation were observed (Supp. Fig. 2). Firstly, very little agonism (changes in baseline) or modulation of the ACh concentration-response curve (changes in pEC_50_ and *E*_max_) were observed if the bottom pendant was merely a methyl group as in **6a**, or a bulkier Boc-protected piperdinylmethyl group such as in **6i**. However, more significant shifts in baseline, potency and maximum responses were observed upon addition of PAMs with the various branched and cyclic aliphatic, tetrahydropyranylmethyl and benzyl groups installed to the pyrazole (compounds **6b-6h**). Together, these results suggest that if the bottom pendant is too small or too large, the allosteric effect is altered, whereas a range of aliphatic, heterocyclic and aromatic functionalities were tolerated. Interestingly, in contrast to these series of compounds, results published by Schubert et al. showed poor tolerability for tetrahydro-2*H*-pyran, benzyl and isobutyl moieties in case of a 4-cyanophenyl top pendant [21]. Consequently, moving forward the cyclopentylmethyl moiety was kept consistent, while investigating other structural features of the allosteric modulators. The direct comparison between compounds **6g** and **7**, indicates that the position of the methyl and/or pyridine nitrogen makes an important contribution to the allosteric profile in this class of PAMs, as analogue **7** showed a noticeable drop in baseline, potency and efficacy compared to **6g**.

Table 2 summarises the changes in baseline, pEC_50_ and *E*_max_ for compounds with alterations to the top pendant. Three different profiles are observed for these compounds (Supp. Fig. 3). All compounds except **6l** and **6u** show evidence of allosteric agonism, as can be seen by the increase in the baseline response. Isoindolin-1-one analogue **6s** is the most potent modulator increasing the potency of ACh (ΔpEC_50_) by over 370-fold. While **6t**, which is structurally closely related to the radio-ligand [^18^F]12 (**1h**), was the second most potent PAM of our series, increasing the potency of ACh 129-fold. Three compounds, containing a phenyl (**6j**), nitroaniline group (**6l**), or an indolin-2-one (**6u**), have very little allosteric agonism, as seen by the low level of changes to the baseline of the ACh concentration-response curves. They also have weak effects on the *E*_max_ of the ACh concentration-response curve. The greatest effect for these three modulators was observed by the increase the pEC_50_ of the ACh concentration response curves in the presence of the allosteric modulators, suggesting these compounds have a tendency towards pure positive allosteric modulation in this functional assay. Interestingly, when comparing **6m** with **6l**, the deletion of the *meta*-nitro group of **6l** resulted in significant increase to the Δbaseline and Δ*E*_max_. Ligands with a *N,N*-dimethylbenzamide (**6o**) or difluorobenzene (**6k**) top pendant are weaker allosteric agonists that did not shift the potency of ACh to a large degree, but afforded significant increases in the ACh *E*_max_. Overall, analogues with a 6-isoindolin-1-one top motif (**6s, 6t**) were the most potent PAMs, however, substantial modifications to the top core were tolerated and led to analogues with a range of allosteric profiles, varying from ago-PAMs to pure PAMs. Noteworthy, modifications to the top pendent resulted in compounds **6k** and **6o** with largest Δ*E*_max_ of the entire series.

Quantification of the changes in baseline, potency and maximum response for the compounds with alterations to the methylpyridine core and the pyrazole moiety are shown in Table 3. The data reveals that the position of the methyl and nitrogen atom on the aromatic core ring seems crucial to the allosteric profile of the synthesised analogues. For instance, the compound **12a**, which only differs by the removal of the methyl group in the 2-position when compared to **1e**, resulted in a significant reduction in the potentiation of ACh (ΔpEC_50_) and decreased direct agonism (Δbaseline). Similarly, **7** (Table 1), **12a-b** and **12e**, which all lack the methyl substituent in position 2 exhibited reduced ability to potentiate the ACh response. Interestingly, when the methyl group was replaced with aromatic nitrogen atom such as in **12c**, both potency and direct agonism were retained. Furthermore, the results highlight that the position of the pyridine nitrogen is crucial, as the ability to potentiate ACh-mediated response curve varies vastly between analogues **12a, 12c** and **12d**. Of note, the different electronic effects of the aromatic heterocycles might also play a role in the compound’s allosteric profiles. Effects on the 1*H*-pyrazole ring were explored with compounds **16** and **20** by substituting it with a triazole moiety. The assessment of changes in the three key parameters of ACh by these compounds showed a decrease the potency, baseline and *E*_max_ of the response.

#### 2.2.2 Rationale for the selection of novel PAMs for full allosteric characterisation

Eight novel analogues were selected for full allosteric quantification based on the results of the initial screening. Compound **1e** was the parent molecule from which the other derivatives were based. Compounds **6c** and **6f** were selected as representative ago-PAMs, with high allosteric agonism (large Δbaseline) and efficacy modulation of Ach (large Δ*E*_max_), but varying abilities to modulate the ACh-mediated response (ΔpEC_50_ **6c** = 1.64 ± 0.20 versus **6f** = 0.73 ± 0.39). Compounds **6k** and **6o** were selected as representative “efficacy” allosteric modulators that potentiate the efficacy of ACh (large Δ*E*_max_) and had little to no direct agonism (no change in baseline). Compounds **6l, 6p, 12a** and **12e** were selected as representative “affinity” allosteric modulators that potentiate the potency of ACh (large ΔpEC_50_) but had little to no direct agonism. The selected compounds were assessed in equilibrium binding assays and two functional assays to determine the affinity (p*K*_B_), cooperativity (*αβ*) values of the modulators, and direct agonist properties (*τ*_B_) with the results shown in Table 4.

#### 2.2.3 Equilibrium binding assay to quantify PAM binding affinity and binding cooperativity with ACh

**Fig. 3:**
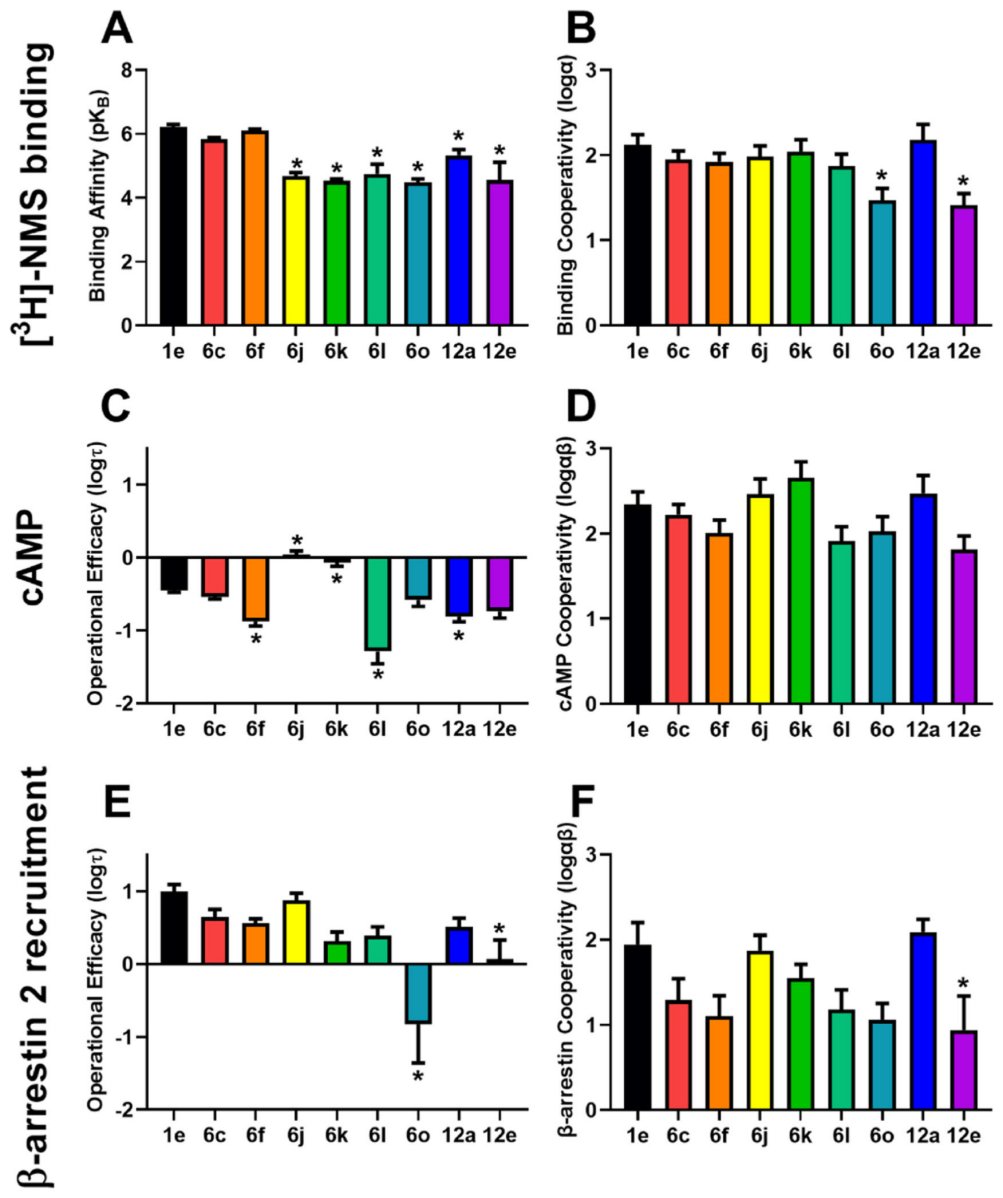
Allosteric parameters calculated for 8 novel allosteric modulators. Full allosteric interaction assays (Supp. Fig. 5) were performed using [^3^H]-*N*-methyl-scopolamine (NMS) (0.5 nM; 6 h, 23 °C) as the radioligand. ACh competed for binding with [^3^H]-NMS at the orthosteric site, which was potentiated in the presence of increasing concentrations of each allosteric modulator. (A) Binding affinity (p*K*_B_) and (B) binding cooperativity with ACh (log *α*_ACh_) values were quantified by fitting the data to the allosteric ternary complex model (equation (2) or (4)) [27,28]. The CAMYEL biosensor was used to measure cAMP signalling (5 min, 37 °C) in full allosteric interaction assays (Supp. Fig. 7). (C) Efficacy (log *τ*_B_) and (D) functional cooperativity with ACh (log *αβ*_ACh_) values were determined by fitting the data to the complete operational model of allosterism and agonism (equation (6)). β-arrestin-eYFP recruitment to the human M_4_ mAChR-Rluc8 receptor was performed in full allosteric interaction assays (Supp. Fig. 8). (E) Efficacy and (F) functional cooperativity with ACh values were determined by fitting the data to the simplified operational model of allosterism and agonism using equation (7). Data were further analysed by performing one-way ANOVA with a Dunnett post-hoc test to compare the parameters of the novel allosteric modulators with **1e**. Data are mean ± SEM of 4–5 independent experiments with repeats in duplicate.

Equilibrium binding assays were performed using [^3^H]-*N*-methyl-scopolamine (NMS) as the radioligand and ACh as the orthosteric ligand in the absence or presence of increasing concentrations of the PAMs for 6 h at 23 °C. The resulting curves were fitted with the allosteric ternary complex model (equation (2) or (4)) [27,28] to determine the affinity and binding cooperativity values for each allosteric modulator (Supp. Figs. 5 and 6). The binding affinities (p*K*_B_) for all ligands were in the micromolar range (1–100 μM) and all modulators increased the affinity of ACh by 30- to 100-fold, consistent with them being PAMs. Compounds **1e, 6c** and **6f** had high negative cooperativity with [^3^H]-NMS, as can be seen by the decrease in the [^3^H]-NMS binding with increasing concentrations of PAMs on the vehicle (Supp. Fig. 5). This effect was not observed with the other selected modulators (Supp. Fig. 5). As can be seen in Fig. 3A, PAMs **6j, 6k, 6l** and **6o**, all had lower affinity values than the parent compound **1e**. These four compounds all had modifications to the top part of the compound compared to **1e**. Significantly lower affinities were also observed with compounds **12a** and **12e**. Compounds **6c** and **6f** with changes to the bottom pendant had similar binding affinity as **1e**. Together these results suggest that both the top part and the core of **1e** are important for allosteric modulator binding to the M_4_ mAChR, whereas the bottom pendant has little effect. Notably, **6j, 6k, 6l, 6o** and **12e** could not be fitted with equation (2) due to not reaching an allosteric ceiling effect at the highest concentration tested (Supp. Fig. 5). The binding data for these compounds was therefore fitted with an alternative analytical model (equation (4)) to estimate the affinity of these ligands (Supp. Fig. 6). The affinity estimates (p*K*_I_) for ACh in the absence or presence of increasing concentrations of PAMs were calculated using a one-site binding isotherm with a Cheng and Prusoff correction (equation (3)). The affinity (p*K*_I_) estimates were plotted against the concentration of PAM and the data fitted with equation (4) to estimate the affinity of the PAMs (Supp. Fig. 6). The affinities derived using equation (4) were then used to fix the p*K*_B_ parameter in equation (2) to determine the binding cooperativity (α) between the orthosteric and allosteric ligands and to fit the curves shown in Supp. Fig. 5. Overall, two of the ligands (**6c, 6f**) maintained a similar profile in terms of affinity for the M_4_ mAChR as the parent compound **1e**, while the remaining ligands had a lower affinity for the M_4_ mAChR than the parent compound **1e**.

Fig. 3B shows the binding cooperativity values estimated by fitting the data to the allosteric ternary complex model (equation (2)). Most of the ligands shown in this figure were found to modulate the affinity of ACh similarly to compound **1e**. Compounds **6o** and **12e** had significantly lower binding cooperativity than **1e**, suggesting that they were not able to modulate the affinity of ACh as strongly as the parent. Compound **6o** had changes to the top pendant, while **12e** had changes to the core, suggesting that changes to either the top or the core of the compound can alter the binding cooperativity between the orthosteric ligand, ACh, and the allosteric modulators. Interestingly, the top pendant of **6o** is a ring opened variant of **1e** (*N,N*-dimethylbenzamide vs 2-methylisoindolin-1-one). This modification reduced the cooperativity from *α* = 131 as observed for compound **1e**, to *α* = 29 for **6o**. It is possible that the isoindoline ring structure plays an important role in forming aromatic interactions with residues of the M_4_ mAChR allosteric site, however other top pendant modifications such as those in **6j, 6k**, and **6l**, which do not have the isoindoline ring as a part of the top pendant, did not follow this trend. Compound **12e**, which possesses a pyrazine core and lacks the methyl substituent present in **1e**, exhibited significant lower binding cooperativity (P = 0.01, one-way ANOVA, Dunnett post-hoc test).

#### 2.2.4 CAMYEL activation to assess PAM direct agonism and functional cooperativity with ACh

FlpInCHO cells transiently expressing the CAMYEL cAMP biosensor were stimulated with agonists and allosteric modulators for 5 min at 37 °C. Bioluminescent resonance energy transfer (BRET) was measured using the LUMIstar and coelenterazine h as the substrate. The concentration-response curves were fitted with the complete operational model of allosterism and agonism (equation (6)) to determine the efficacy (*τ*_B_) and functional cooperativity (log *αβ*) values for the nine allosteric modulators. Compounds **1e, 6j, 6k, 12a** all showed direct allosteric agonism activity, increasing intracellular cAMP in their own rights from the allosteric site. This can be seen in Supp. Fig. 7 as an increase in the baseline of the concentration-response curves, consistent with our initial screening experiments. Notably, the allosteric agonism was reduced for compounds **6o, 6l, 12e** compared to **1e**. The operational efficacies of **6j** (*τ*_B_ = 1.1) and **6k** (*τ*_B_ = 0.9) were greater than compound **1e** (*τ*_B_ = 0.4) in increasing intracellular cAMP (Fig. 3C). The changes in compounds **6j** and **6k** were at the top of the molecule, where both compounds had smaller top pendants than **1e**. This suggests that fewer interactions between the top pendant and the M_4_ mAChR results in stronger allosteric operational efficacy. There is no structure of compound **1e** with the M_4_ mAChR and no mutagenesis has been performed to date to determine how **1e** engages with the allosteric pocket, however, it potentially binds to the “common” allosteric site that lies directly above the tyrosine lid and is used by other M_4_ PAMS, such as LY2033298 and VU0467154 [29]. Further structural biology studies to determine the structure of the M_4_ mAChR with compound **1e**, are required to understand the possible interactions involved in driving allosteric efficacy by **1e** and the related compounds **6j** and **6k**. Compounds **6f** (*τ*_B_ = 0.1), **6l** (*τ*_B_ = 0.05) and **12a** (*τ*_B_ = 0.1) had significantly lower operational efficacy than **1e**. The changes to compounds **6f** (bottom), **6l** (top) and **12a** (core) were in different locations, making it difficult to determine the reason for the decreased efficacy compared to **1e**. Nevertheless, these changes all diminished direct efficacy compared to **1e**, possibly due to alterations in the ligand conformation or charges associated with the compounds that cause these ligands to bind to the M_4_ mAChR to stabilise slightly different conformations of the receptor.

**Fig. 4:**
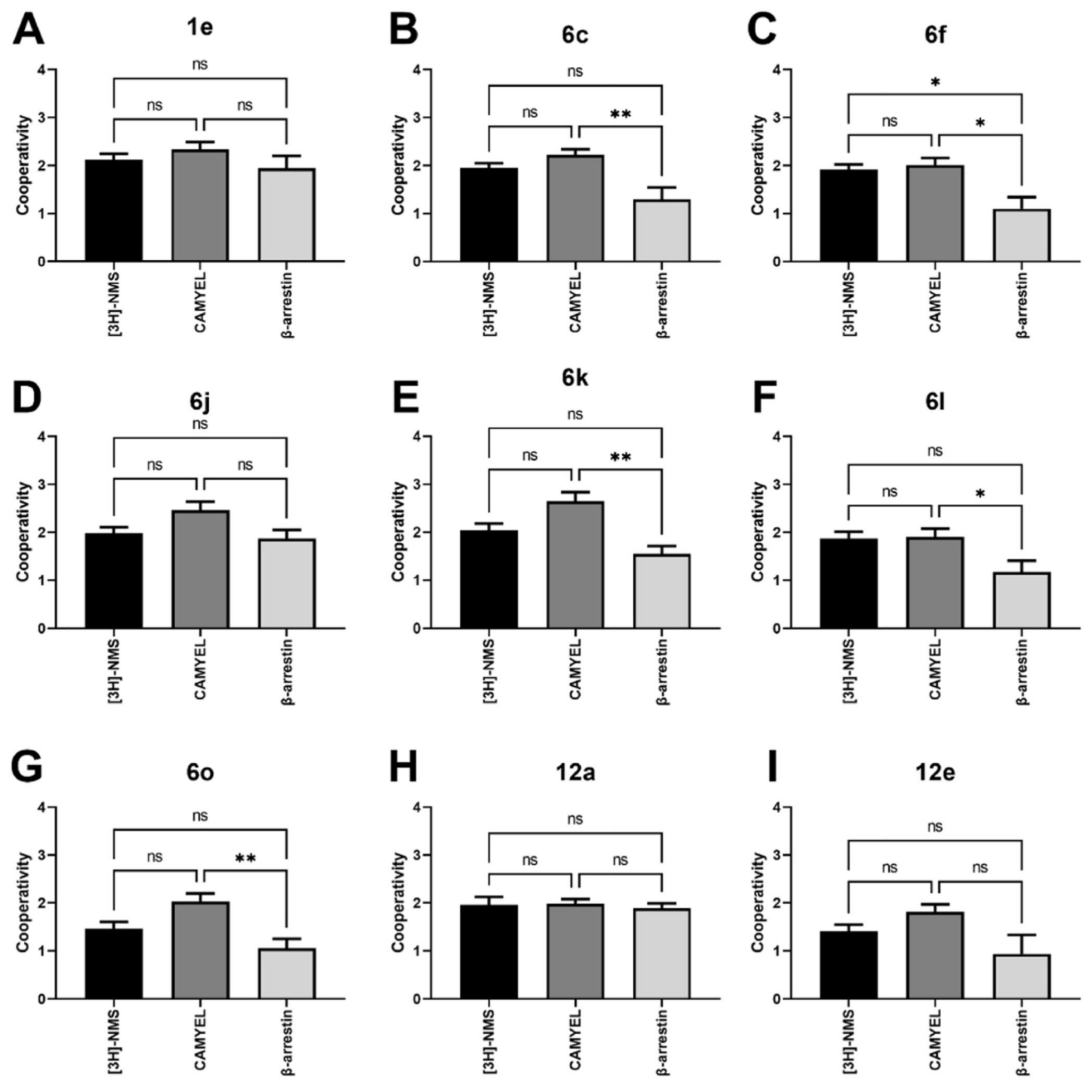
Comparison of cooperativity parameters determined from binding and functional assays. The cooperativity parameters determined by fitting the full allosteric interaction assays (Supplementary Figs. 5–8) with the allosteric models were plotted for each allosteric modulator. (A) **1e**, (B) **6c**, (C) **6f**, (D) **6j**, (E) **6k**, (F) **6l**, (G) **6o**, (H) **12a**, (I) **12e**. Data were analysed by one-way ANOVA and a Tukey’s post-hoc test to compare all groups with each other. Data are mean ± SEM of 4–5 experiments with repeats in duplicate.

It should be noted that the cAMP pathway measured is an activation of the CAMYEL biosensor [30] driven by increases in cAMP levels [24–26]. When looking at the functional cooperativity of the compounds in Fig. 3D, none of the compounds had significant differences in functional cooperativity compared to the parent compound **1e**. Comparing the binding cooperativity with the functional cooperativity for cAMP in Fig. 4 shows that there were no significant differences between the cooperativity estimates, suggesting that compound **1e** and the new derivatives synthesised in this study are all affinity modulators of ACh at the M_4_ mAChR.

#### 2.2.5 β-Arrestin 2 recruitment to assess PAM direct efficacy and functional cooperativity with ACh

FlpInCHO cells transiently co-expressing eYFP-β-arrestin 2 and M_4_ mAChR-Rluc8 were stimulated with agonists and allosteric modulators for 5 min at 37 °C. BRET was measured using the LUMIstar and coelenterazine h as the substrate. The concentration-response curves were fitted with the simplified operational model of allosterism and agonism (equation (7)), to determine the efficacy (*τ*_B_) and functional cooperativity (log *αβ*) values for the nine allosteric modulators. Allosteric agonism was observed for **1e, 6c, 6f, 6j, 6l** and **12a** as can be seen by the increase in the baseline for the ACh concentration-response curves in the presence of increasing concentrations of PAMs in Supp. Fig. 8. These PAMs are based on structural modifications to the bottom (**6c** and **6f**), top (**6j** and **6l**) and methyl pyridine core (**12a**) showing that no single region of the parent molecule is solely responsible for determining whether an M_4_ PAM with a 2-phenyl-3-(1*H*-pyrazol-4-yl)pyridine will have direct allosteric agonism or not. Likewise, very little operational efficacy towards *β-*arrestin 2 recruitment was observed by compounds with changes to the bottom (**6o**) and core motif (**12a**), suggesting that there are multiple alternatives to reduce allosteric agonism (Fig. 3E). The functional cooperativity estimates in β-arrestin recruitment were not significantly different to the parent compound **1e** (Fig. 3F). Compound **12e** with changes to the core of the molecule was the exception, with significantly lower functional cooperativity for β-arrestin 2 recruitment to the M_4_ mAChR compared to **1e**. Comparing the functional cooperativity values calculated for β-arrestin 2 recruitment with the affinity cooperativity estimates calculated from [^3^H]-NMS binding no significant differences were observed for any compound as shown in Fig. 4. This suggests that the parent compound **1e**, and all derivative synthesised are affinity modulators of ACh at the M_4_ mAChR.

### 2.3 Assessing the potential for CNS exposure of selected M_4_ PAMs

As a prudent precursor to future *in vivo* evaluation in rodent behavioural models, a probative exposure study was conducted in mice for a small subset of molecules in order to the establish whether they are able to cross the blood brain barrier and enter the brain parenchyma. Initial *in vivo* exposure of compound **1e** showed very low concentrations of this compound remained in the brain and plasma 90 min post-dose (Table 5). This finding was consistent with the online metabolism predictive tool, pkCSM which predicted a total clearance of 9.9 ml/min/kg [31]. Compounds **6k** and **6l** were chosen for this study based on calculations using pkCSM, which predicted lower clearance (CL_tot_: **6k** = 2.5 ml/min/kg; **6l** = 2.1 ml/min/kg) of these compounds relative to compound **1e**. Following IP administration, concentrations in brain and plasma were determined at 20 and 90 min as this is the timeframe relevant to acute *in vivo* behavioural testing [32] and the brain-to-plasma partition ratio (*K*_p_ = C_brain_/C_plasma_) was determined (Table 5). All three compounds exhibited *K*_p_ values that exceeded the lower limit expected for a non-CNS penetrant molecule (i.e. one that resides solely within the brain vasculature; *K*_p_ – 0.017 in C57Bl6 mice [33]), indicating that all three compounds could successfully cross the blood brain barrier and achieve measurable exposure within the CNS.

### 2.4 Extracellular signal-regulated kinase 1/2 (ERK1/2) phosphorylation to assess PAM selectivity

ERK1/2 proteins belong to the mitogen-activated protein kinase (MAPK) family, which plays a critical role in signalling cascades and transmits extracellular signals to intracellular targets, and in particular in cell proliferation, differentiation, apoptosis, angiogenesis and tumour metastasis than other pathways. Upon agonist activation, most GPCR can induce ERK1/2 phosphorylation. Indeed, activation of any of the 5 mAChRs (M_1_-M_5_) lead to ERK1/2 phosphorylation [5,34], making this assay powerful for assessment of subtype selectivity. To assess the degree of selectivity of **1e, 6k** and **6l** across all mAChR, we performed concentration-response curves with ACh in absence of presence of 10 μM of each of the selected PAM (Supp. Fig. 9). Except for **1e** at the M_2_ mAChR, no allosteric effect was observed at M_1_-M_3_ and M_5_ mAChRs. The observed allosteric effect of all 3 selected PAMs at the M_4_ mAChR in ERK1/2 phosphorylation was consistent with the effects quantified in CAMYEL and *β*-arrestin recruitment.

## 3 Conclusion

The development of drugs targeting M_4_ mAChRs has remained challenging partially due to the high homology of the orthosteric binding site across receptor subtypes resulting in drugs with poor subtype selectivity and adverse effect profiles. The design of allosteric ligands has provided ligands with significantly improved mAChR subtype selectivity, yet species variability and poor physicochemical properties has limited their further development for *in vivo* validation. Additionally, as we show here, allosteric ligands exhibit complex signalling profiles when assessed across the range of signalling pathways that these receptors can couple to, and it remains unclear what signalling profile result to a preferential therapeutic outcome. Hence, establishing SAR of allosteric modulators across more than one signalling pathway is critical to further our understanding of their mechanism of action and unsure the development of allosteric ligands with improved physicochemical properties.

Here, we synthesised and pharmacologically evaluated 28 PAMs structurally related to the previously published M_4_ mAChRs PAMs **1e**, Me–C-c, [^11^C]MK-6884 and [^18^F]12. Our initial screening in cAMP highlighted that changes to the bottom pendant were generally well tolerated, unless a small or bulkier substituent was installed. Whereas changes to the core and top section afforded variable results and no clear trends were identified due to the flat SAR. This highlights the need to further improve our understating of the key ligand-receptor interactions at the M_4_ mAChR receptor, which can assist the development of allosteric ligands with desired allosteric profiles.

Full characterisation of selected compounds with different allosteric profiles was performed for 8 novel ligands. Compounds **6k** and **6l** were chosen for preliminary *in vivo* exposure studies based on their binding affinity, selectivity profile and predictive calculations using the online metabolism prediction tool, pkCSM. Both PAMs exhibited improved blood-brain barrier penetration and longer drug exposure compared to **1e** and promise to be useful tool compounds for *in vivo* studies in the brain.

## 4 Experimental section

### 4.1 General chemistry methods

Chemicals and solvents were purchased from standard suppliers and used without further purification. Davisil® silica gel (40–63 μm) for flash column chromatography was supplied by Grace Davison Discovery Sciences (Victoria, Australia) and deuterated solvents were purchased from Cambridge Isotope Laboratories, Inc. (USA, distributed by Novachem Pty Ltd, Victoria, Australia).

Reactions were monitored by thin layer chromatography on commercially available precoated aluminium-backed plates (Merck Kieselgel 60 F_254_). Visualisation was by examination under UV light (254 and 366 nm), curcumin and/or permanganate stain. Organic solvents were evaporated *in vacuo* at ≤40 °C (water bath temperature).

^1^H NMR spectra were recorded on a Bruker Avance Nanobay III 400 MHz Ultrashield Plus spectrometer at 400.13 MHz. Chemical shifts (*δ*) are recorded in parts per million (ppm) with reference to the chemical shift of the deuterated solvent. Coupling constants (*J*) are recorded in Hz and the significant multiplicities described by singlet (s), broad singlet (br s), doublet (d), triplet (t), quadruplet (q), broad (br), multiplet (m), doublet of doublets (dd), doublet of triplets (dt) and doublet of doublet of doublets (ddd). DEPTQ spectra were recorded to identify all carbon signals and discriminate between CH/CH_3_ and C_q_/CH_2_ signals.

LCMS were run to verify reaction outcome and purity using an Agilent 6120 Series Single Quad coupled to an Agilent 1260 Series HPLC. The following buffers were used; buffer A: 0.1% formic acid in H_2_O; buffer B: 0.1% formic acid in MeCN. The following gradient was used with a Poroshell 120 EC-C18 50 × 3.0 mm 2.7 μm column, and a flow rate of 0.5 mL/min and total run time of 5 min; 0–1 min 95% buffer A and 5% buffer B, from 1 to 2.5 min up to 0% buffer A and 100% buffer B, held at this composition until 3.8 min, 3.8–4 min 95% buffer A and 5% buffer B, held until 5 min at this composition. Mass spectra were acquired in positive and negative ion mode with a scan range of 100–1000 *m/z*. UV detection was carried out at 214 and 254 nm. All retention times (*t*_R_) are quoted in minutes. Preparative HPLC was performed using an Agilent 1260 infinity coupled with a binary preparative pump and Agilent 1260 FC-PS fraction collector, using Agilent OpenLAB CDS software (Rev C.01.04), and an Agilent 7 μM XDB-C8 21.2 × 250 mm column. The following buffers were used unless stated otherwise: buffer A was H_2_O; buffer B was MeCN, with sample being run at a gradient of 5% or 30% buffer B to 100% buffer B over 10 min, at a flow rate of 20 mL/min. All screening compounds were of >95% purity unless stated otherwise.

### 4.2 Chemistry

#### 4.2.1 General procedure A: alkylation of 4-pyrazoleboronic acid pinacol ester

The respective halide (1.25 equiv.), K_2_CO_3_ (2.0 equiv.) and 4-pyrazoleboronic acid pinacol ester (1.0 equiv.) were stirred in DMF (3 mL/100 mg) at 60 °C until the reaction appeared complete or stagnate (reaction progress was monitored via TLC). The reaction mixture was diluted with EtOAc and washed with water (2 × 50 mL) and brine (50 mL). The organic layer was dried with Na_2_SO_4_, filtered and concentrated under reduced pressure.

#### 4.2.2 General procedure B: Suzuki reaction conditions 1

A mixture of the aryl halide (1.0 equiv.), Cs_2_CO_3_ (1.5 equiv.) and the respective boronic acid or pinacol ester (1.5 equiv.) in dry DMF or DME (3 mL/100 mg) was flushed with nitrogen. PdCl_2_(PPh_3_)_2_ (0.1 equiv.) was added and the reaction mixture, which was stirred at 85–100 °C until full conversation of starting material was observed (reaction progress was monitored via LC-MS). The reaction mixture was diluted with EtOAc and washed with water (2 × 50 mL) and brine (50 mL). The organic layer was dried with Na_2_SO_4_, filtered and concentrated under reduced pressure.

#### 4.2.3 General procedure C: Suzuki reaction conditions 2

A mixture of respective aryl halide (1.0 equiv.) and appropriate boronic acid or pinacol ester (1.5 equiv.) in degassed THF/1 M Na_2_CO_3 (aq)_ (3 mL/100 mg) was flushed with nitrogen. PdCl_2_(PPh_3_)_2_ (0.1 equiv.) was added and the reaction mixture stirred under reflux until full conversion of the starting material was observed (reaction progress was monitored by LC-MS). The THF was evaporated under reduced pressure. The residue was dissolved in EtOAc and washed with water (2 × 50 mL) and brine (50 mL). The organic layer was dried with Na_2_SO_4_, filtered and concentrated under reduced pressure.

#### 4.2.4 6-(3-(1-(Cyclopentylmethyl)-1H-pyrazol-4-yl)-6-methylpyridin-2-yl)-2-methylisoindolin-1-one (**1e**)

Synthesised according to General Procedure B. The crude product was purified by FCC (PE: EtOAc 8:2 → EtOAc 100% → EtOAc: MeOH 9:1), followed by preparative HPLC (30–100%) to give the title compound as a colourless oil (4 mg, 6%). ^1^H NMR (CDCl_3_) *δ* 7.94 (d, *J* = 0.8 Hz, 1H), 7.71 (d, *J* = 7.9 Hz, 1H), 7.63–7.56 (m, 1H), 7.42 (d, *J* = 7.8 Hz, 1H), 7.27 (s, 1H), 7.22 (d, *J* = 8.0 Hz, 1H), 6.90 (s, 1H), 4.41 (s, 2H), 3.89 (d, *J* = 7.5 Hz, 2H), 3.23 (s, 3H), 2.66 (s, 3H), 2.35–2.24 (m, 1H), 1.66–1.50 (m, 6H), 1.16–1.04 (m, 2H); ^13^C NMR (CDCl_3_) *δ* 168.3, 156.3, 155.5, 141.3, 140.5, 138.2, 137.5, 133.1, 132.5, 128.2, 124.8, 124.4, 122.4 (2 ×), 119.4, 57.0, 51.9, 40.7, 30.1, 29.5, 24.9, 24.2; *m/z* MS (TOF ES^+^) 387.0 [M - H]^+^; LC-MS *t*_R_: 3.06; HRMS - C_24_H_27_N_4_O [M+H]^+^ calcd 387.2185; found 387.2187.

#### 4.2.5 1-(Cyclopropylmethyl)-4-(4,4,5,5-tetramethyl-1,3,2-dioxaborolan-2-yl)-1H-pyrazole (**3b**)

Synthesised according to General Procedure A. The crude product was purified by FCC (PE 100%→ PE: EtOAc 7:3) to afford the title compound as a colourless oil (411 mg, 32%). ^1^H NMR (CDCl_3_) *δ* 7.74 (s, 1H), 7.72 (s, 1H), 3.93 (d, *J* = 7.1 Hz, 2H), 1.25 (s, 12H), 1.23–1.18 (m, 1H), 0.61–0.54 (m, 2H), 0.33–0.27 (m, 2H).

#### 4.2.6 Isobutyl-4-(4,4,5,5-tetramethyl-1,3,2-dioxaborolan-2-yl)-1H-pyrazole (**3c**)

Synthesised according to General Procedure A. The crude product was purified by FCC (PE 100%→ PE: EtOAc 7:3) to afford the title compound as a colourless oil (744 mg, 58%). ^1^H NMR (CDCl_3_) *δ* 7.77 (s, 1H), 7.64 (s, 1H), 3.90 (d, *J* = 7.2 Hz, 2H), 2.27–2.14 (m, 1H), 1.31 (s, 12H), 0.88 (d, *J* = 6.7 Hz, 6H).

#### 4.2.7 1-(Cyclobutylmethyl)-4-(4,4,5,5-tetramethyl-1,3,2-dioxaborolan-2-yl)-1H-pyrazole (**3d**)

Synthesised according to General Procedure A. The crude product was purified by FCC (PE 100%→ PE: EtOAc 6:4) to afford the title compound as a colourless oil (791 mg, 59%). ^1^H NMR (CDCl_3_) *δ* 7.74 (s, 1H), 7.62 (s, 1H), 4.11 (d, *J* = 7.3 Hz, 2H), 2.86–2.72 (m, 1H), 2.10–1.98 (m, 2H), 1.95–1.70 (m, 4H), 1.29 (s, 12H).

#### 4.2.8 2-Chloro-3-(1-(cyclopentylmethyl)-1H-pyrazol-4-yl)-6-methylpyridine (**3e**)

Synthesised according to General Procedure A. The crude product was purified by FCC (PE 100% → PE: EtOAc 1:1) to afford the title compound as a light-yellow oil (152 mg, 46%). ^1^H NMR (CDCl_3_) *δ* 7.81 (d, *J* = 0.5 Hz, 1H), 7.73 (d, *J* = 0.6 Hz, 1H), 7.63 (d, *J* = 7.8 Hz, 1H), 7.04 (d, *J* = 7.9 Hz, 1H), 4.03 (d, *J* = 7.5 Hz, 2H), 2.47 (s, 3H), 2.46–2.38 (m, 1H), 1.75–1.46 (m, 6H), 1.29–1.20 (m, 2H); *m/z* MS (TOF ES^+^) 276.0 [M – H]^+^; LC-MS *t*_R_: 3.45.

#### 4.2.9 1-(Cyclohexylmethyl)-4-(4,4,5,5-tetramethyl-1,3,2-dioxaborolan-2-yl)-1H-pyrazole (**3f**)

Synthesised according to General Procedure A. The crude product was purified by FCC (PE 100%→ PE: EtOAc 6:4) to afford the title compound as a colourless oil (1.02 g, 68%). ^1^H NMR (CDCl_3_) *δ* 7.77 (s, 1H), 7.62 (s, 1H), 3.93 (d, *J* = 7.1 Hz, 2H), 1.93–1.81 (m, 1H), 1.73–1.56 (m, 5H), 1.36–1.26 (m, 12H), 1.22–1.12 (m, 3H), 0.97–0.89 (m, 2H).

#### 4.2.10 1-((Tetrahydro-2H-pyran-4-yl)methyl)-4-(4,4,5,5-tetramethyl-1,3,2-dioxaborolan-2-yl)-1H-pyrazole (**3g**)

Synthesised according to General Procedure A. The crude product was purified by FCC (PE 100%→ PE: EtOAc 1:1) to afford the title compound as a colourless oil (617 mg, 41%). ^1^H NMR (CDCl_3_) *δ* 7.72 (s, 1H), 7.58 (s, 1H), 3.92 (d, *J* = 7.2 Hz, 2H), 3.91–3.85 (m, 2H), 3.27 (td, *J* = 11.8, 2.2 Hz, 2H), 2.15–2.02 (m, 1H), 1.43–1.37 (m, 2H), 1.25 (s, 12H).

#### 4.2.11 1-Benzyl-4-(4,4,5,5-tetramethyl-1,3,2-dioxaborolan-2-yl)-1H-pyrazole (**3h**)

Synthesised according to General Procedure A. The crude product was purified by FCC (PE 100%→ PE: EtOAc 1:1) to afford the title compound as a colourless oil (501 mg, 34%). ^1^H NMR (CDCl_3_) *δ* 7.87–7.81 (m, 1H), 7.69 (s, 1H), 7.39–7.31 (m, 3H), 7.27–7.22 (m, 2H), 5.33 (s, 2H), 1.32 (s, 12H); *m/z* MS (TOF ES^+^) 285.2; LC-MS *t*_R_: 4.07.

#### 4.2.12 tert-Butyl 4-((4-(4,4,5,5-tetramethyl-1,3,2-dioxaborolan-2-yl)-1H-pyrazol-1-yl)methyl)-piperidine-1-carboxylate (**3i**)

Synthesised according to General Procedure A. The crude product was purified by FCC (PE 100%→ PE: EtOAc 1:1) to afford the title compound as a light yellowish oil (2.16 g, 54%). ^1^H NMR (CDCl_3_) *δ* 7.80 (s, 1H), 7.65 (s, 1H), 4.16–4.06 (m, 2H), 4.01 (d, *J* = 7.1 Hz, 2H), 2.67 (br t, *J* = 12.4 Hz, 2H), 2.15–2.06 (m, 1H), 1.60–1.51 (m, 2H), 1.46 (s, 9H), 1.33 (s, 12H), 1.21–1.11 (m, 2H).

#### 4.2.13 2-Chloro-6-methyl-3-(1-methyl-1H-pyrazol-4-yl)pyridine (**5a**)

Synthesised according to General Procedure A. The crude product was purified by FCC (PE 100% → EtOAc 100%) to afford the title compound as a yellow oil (288 mg, 29%). ^1^H NMR (CDCl_3_) *δ* 7.78 (s, 1H), 7.75–7.70 (m, 1H), 7.61 (d, *J* = 7.8 Hz, 1H), 7.05 (d, *J* = 7.8 Hz, 1H), 3.91 (s, 3H), 2.48 (s, 3H); *m/z* MS (TOF ES^+^) 208.1 [M – H]^+^; LC-MS *t*_R_: 2.48.

#### 4.2.14 2-Chloro-3-(1-isobutyl-1H-pyrazol-4-yl)-6-methylpyridine (**5b**)

Synthesised according to General Procedure B. The crude product was purified by FCC (PE 100% → PE: EtOAc 1:1) to afford the title compound as a colourless oil (164 mg, 22%). ^1^H NMR (CDCl_3_) *δ* 7.80 (d, *J* = 0.5 Hz, 1H), 7.75 (d, *J* = 0.6 Hz, 1H), 7.64 (d, *J* = 7.8 Hz, 1H), 7.08–7.04 (m, 1H), 3.93 (d, *J* = 7.3 Hz, 2H), 2.50 (s, 3H), 2.27–2.16 (m, 1H), 0.91 (d, *J* = 6.7 Hz, 6H); *m/z* MS (TOF ES^+^) 250.1; LC-MS *t*_R_: 3.43.

#### 4.2.15 2-Chloro-3-(1-(cyclopropylmethyl)-1H-pyrazol-4-yl)-6-methylpyridine (**5c**)

Synthesised according to General Procedure B. The crude product was purified by FCC (PE 100% → PE: EtOAc 1:1) to afford the title compound as a light-yellow oil (140 mg, 34%). ^1^H NMR (CDCl_3_) *δ* 7.93–7.88 (m, 1H), 7.75 (d, *J* = 0.7 Hz, 1H), 7.63 (d, *J* = 7.8 Hz, 1H), 7.05 (d, *J* = 7.8 Hz, 1H), 3.99 (d, *J* = 7.1 Hz, 2H), 2.48 (s, 3H), 1.33–1.24 (m, 1H), 0.67–0.59 (m, 2H), 0.40–0.33 (m, 2H); *m/z* MS (TOF ES^+^) 248.0 [M – H]^+^; LC-MS *t*_R_: 4.29.

#### 4.2.16 2-Chloro-3-(1-(cyclobutylmethyl)-1H-pyrazol-4-yl)-6-methylpyridine (**5d**)

Synthesised according to General Procedure B. The crude product was purified by FCC (PE 100% → PE: EtOAc 1:1), followed by FCC (DCM 100% → DCM: MeOH 9:1) to afford the title compound as a colourless oil (278 mg, 35%). ^1^H NMR (CDCl_3_) *δ* 7.81 (d, *J* = 0.6 Hz, 1H), 7.75 (d, *J* = 0.6 Hz, 1H), 7.65 (d, *J* = 7.8 Hz, 1H), 7.07 (d, *J* = 7.8 Hz, 1H), 4.15 (d, *J* = 7.3 Hz, 2H), 2.90–2.78 (m, 1H), 2.50 (s, 3H), 2.13–2.03 (m, 2H), 1.94–1.75 (m, 4H); *m/z* MS (TOF ES^+^) 262.1; LC-MS *t*_R_: 2.99.

#### 4.2.17 2-Chloro-3-(1-(cyclopentylmethyl)-1H-pyrazol-4-yl)-6-methylpyridine (**5e**)

Synthesised according to General Procedure B. The crude product was purified by FCC (PE 100% → PE: EtOAc 1:1) to give the title compound as a light-yellow oil (152 mg, 46%). ^1^H NMR (CDCl_3_) *δ* 7.81 (d, *J* = 0.5 Hz, 1H), 7.73 (d, *J* = 0.6 Hz, 1H), 7.63 (d, *J* = 7.8 Hz, 1H), 7.04 (d, *J* = 7.9 Hz, 1H), 4.03 (d, *J* = 7.5 Hz, 2H), 2.47 (s, 3H), 2.46–2.38 (m, 1H), 1.75–1.46 (m, 6H), 1.29–1.20 (m, 2H); *m/z* MS (TOF ES^+^) 276.0 [M – H]^+^; LC-MS *t*_R_: 3.45.

#### 4.2.18 2-Chloro-3-(1-(cyclohexylmethyl)-1H-pyrazol-4-yl)-6-methylpyridine (**5f**)

Synthesised according to General Procedure B. The crude product was purified by FCC (PE 100% → PE: EtOAc 1:1) to afford the title compound as a light-yellow colourless oil (274 mg, 26%). ^1^H NMR (CDCl_3_) *δ* 7.75 (s, 1H), 7.71 (s, 1H), 7.61 (d, *J* = 7.8 Hz, 1H), 7.02 (d, *J* = 7.8 Hz, 1H), 3.91 (d, *J* = 7.2 Hz, 2H), 2.46 (s, 3H), 1.89–1.78 (m, 1H), 1.70–1.53 (m, 5H), 1.21–1.06 (m, 3H), 0.97–0.84 (m, 2H); *m/z* MS (TOF ES^+^) 290.0 [M – H]^+^; LC-MS *t*_R_: 4.73.

#### 4.2.19 2-Chloro-6-methyl-3-(1-((tetrahydro-2H-pyran-4-yl)methyl)-1H-pyrazol-4-yl)pyridine (**5g**)

Synthesised according to General Procedure B. The crude product was purified by FCC (PE 100% → PE: EtOAc 1:1), followed by FCC (DCM 100% → DCM: MeOH 9:1) to afford the title compound as a colourless oil (77 mg, 12%). The product contained some 3-iodo-6-methyl-2-(1-((tetrahydro-2*H*-pyran-4-yl)methyl)-1*H*-pyrazol-4-yl)pyridine impurity, which was carried forward to the next step. ^1^H NMR (CDCl_3_) *δ* 7.76 (d, *J* = 0.5 Hz, 1H), 7.72 (d, *J* = 0.5 Hz, 1H), 7.60 (d, *J* = 7.8 Hz, 1H), 7.03 (d, *J* = 7.9 Hz, 1H), 3.96 (d, *J* = 7.2 Hz, 2H), 3.93–3.86 (m, 2H), 3.33–3.23 (m, 2H), 2.46 (s, 3H), 2.18–2.07 (m, 1H), 1.50–1.43 (m, 2H), 1.38–1.24 (m, 2H); *m/z* MS (TOF ES^+^) 292.1; LC-MS *t*_R_: 2.61.

#### 4.2.20 3-(1-Benzyl-1H-pyrazol-4-yl)-2-chloro-6-methylpyridine (**5h**)

Synthesised according to General Procedure B. The crude product was purified by FCC (PE 100%→ PE: EtOAc 8:2) to afford the title compound as a light-yellow oil (192 mg, 42%). ^1^H NMR (CDCl_3_) *δ* 7.76 (d, *J* = 2.2 Hz, 2H), 7.56 (d, *J* = 7.8 Hz, 1H), 7.30–7.14 (m, 5H), 6.99 (d, *J* = 7.9 Hz, 1H), 5.26 (s, 2H), 2.43 (s, 3H); *m/z* MS (TOF ES^+^) 284.0; LC-MS *t*_R_: 4.03.

#### 4.2.21 tert-Butyl 4-((4-(2-chloro-6-methylpyridin-3-yl)-1H-pyrazol-1-yl) methyl)piperidine-1-carboxylate (**5i**)

Synthesised according to General Procedure B. The crude product was purified by FCC (PE: EtOAc 8:2 → PE: EtOAc 4:6) to afford the title compound as a light-yellow oil (841 mg, 44%). ^1^H NMR (CDCl_3_) *δ* 7.81 (d, *J* = 0.5 Hz, 1H), 7.79 (d, *J* = 0.5 Hz, 1H), 7.67 (d, *J* = 7.8 Hz, 1H), 7.10 (d, *J* = 7.8 Hz, 1H), 4.18–4.06 (m, 2H), 4.03 (d, *J* = 7.2 Hz, 2H), 2.68 (br t, *J* = 12.2 Hz, 2H), 2.53 (s, 3H), 2.12–2.06 (m, 1H), 1.62–1.55 (m, 2H), 1.44 (s, 9H), 1.21–1.16 (m, 2H); *m/z* MS (TOF ES^+^) no ioni-zation; LC-MS *t*_R_: 3.41.

#### 4.2.22 2-Methyl-6-(6-methyl-3-(1-methyl-1H-pyrazol-4-yl)pyridin-2-yl) isoindolin-1-one (**6a**)

Synthesised according to General Procedure B. The crude product was purified by FCC (PE 100% → EtOAc 100% → DCM 100% → DCM: MeOH 9:1), followed by preparative HPLC (5–100%) to afford the title compound as a white resin (15 mg, 10%). ^1^H NMR (CDCl_3_) *δ* 7.90 (d, *J* = 1.0 Hz, 1H), 7.62 (d, *J* = 7.9 Hz, 1H), 7.56 (dd, *J* = 7.8, 1.6 Hz, 1H), 7.39 (dd, *J* = 7.8, 0.5 Hz, 1H), 7.15 (d, *J* = 7.9 Hz, 1H), 7.10 (d, *J* = 0.6 Hz, 1H), 7.02 (s, 1H), 4.39 (s, 2H), 3.78 (s, 3H), 3.19 (s, 3H), 2.59 (s, 3H); ^13^C NMR (CDCl_3_) *δ* 168.4, 156.4, 155.5, 141.3, 140.5, 138.6, 137.5, 133.0, 132.5, 128.8, 124.7, 124.1, 122.5, 122.3, 120.1, 51.9, 39.0, 29.5, 24.3; *m/z* MS (hydrophobic method) (TOF ES^+^) 319.1 [M – H]^+^; LC-MS *t*_R_: 2.46; HRMS - C_19_H_19_N_4_O [M+H]^+^ calcd 319.1559; found 319.1560.

#### 4.2.23 6-(3-(1-Isobutyl-1H-pyrazol-4-yl)-6-methylpyridin-2-yl)-2-methylisoindolin-1-one (**6b**)

Synthesised according to General Procedure B. The crude product was purified by FCC (EtOAc: PE 1:1 → EtOAc 100% → DCM → DCM: MeOH 9:1), followed by preparative HPLC (5–100%) to afford the title compound as a colourless oil (33 mg, 40%). ^1^H NMR (CDCl_3_) *δ* 7.92 (d, *J* = 1.0 Hz, 1H), 7.64 (d, *J* = 7.9 Hz, 1H), 7.53 (dd, *J* = 7.8, 1.6 Hz, 1H), 7.35 (dd, *J* = 7.8, 0.4 Hz, 1H), 7.24–7.21 (m, 1H), 7.16 (d, *J* = 8.0 Hz, 1H), 6.88 (d, *J* = 0.4 Hz, 1H), 4.37 (s, 2H), 3.74 (d, *J* = 7.3 Hz, 2H), 3.19 (s, 3H), 2.59 (s, 3H), 2.11–1.99 (m, 1H), 0.79 (d, *J* = 6.7 Hz, 6H); ^13^C NMR (CDCl_3_) *δ* 168.3, 156.4, 155.5, 141.3, 140.5, 138.4, 137.5, 133.1, 132.5, 128.6, 124.8, 124.3, 122.3 (2 ×), 119.4, 59.7, 51.9, 29.5 (2 ×), 24.3, 19.8; *m/z* MS (TOF ES^+^) hydrophobic 361.2; LC-MS *t*_R_: 2.95; HRMS - C_22_H_25_N_4_O [M+H]^+^ calcd 361.2028; found 361.2030.

#### 4.2.24 6-(3-(1-(Cyclopropylmethyl)-1H-pyrazol-4-yl)-6-methylpyridin-2-yl)-2-methylisoindolin-1-one (**6c**)

Synthesised according to General Procedure B. The crude product was purified by FCC (EtOAc: PE 2:8 → EtOAc 100% → DCM 100% → DCM: MeOH 9:1), followed by preparative HPLC (5–100%) to afford the title compound as a colourless oil (16 mg, 14%). ^1^H NMR (CDCl_3_) *δ* 7.92 (s, 1H), 7.66 (d, *J* = 7.8 Hz, 1H), 7.56 (d, *J* = 7.5 Hz, 1H), 7.39 (d, *J* = 7.6 Hz, 1H), 7.19 (s, 1H), 7.16 (d, *J* = 7.8 Hz, 1H), 7.05 (s, 1H), 4.38 (s, 2H), 3.83 (d, *J* = 7.1 Hz, 2H), 3.19 (s, 3H), 2.60 (s, 3H), 1.20–1.09 (m, 1H), 0.56–0.49 (m, 2H), 0.23–0.18 (m, 2H); ^13^C NMR (CDCl_3_) *δ* 168.3, 156.3, 155.4, 141.3, 140.5, 138.2, 137.4, 133.0, 132.6, 127.6, 124.8, 124.4, 122.4 (2 ×), 119.6, 56.8, 51.9, 29.5, 24.2, 11.0, 3.9; *m/z* MS (TOF ES^+^) 359.0 [M – H]^+^; LC-MS *t*_R_: 3.28; HRMS - C_22_H_23_N_4_O [M+H]^+^ calcd 359.1872; found 359.1878.

#### 4.2.25 6-(3-(1-(Cyclobutylmethyl)-1H-pyrazol-4-yl)-6-methylpyridin-2-yl)-2-methylisoindolin-1-one (**6d**)

Synthesised according to General Procedure B. The crude product was purified by FCC (EtOAc: PE 1:1 → EtOAc 100% → DCM → DCM: MeOH 9:1), followed by preparative HPLC (5–100%) to afford the title compound as a yellow oil (15 mg, 21%). ^1^H NMR (CDCl_3_) *δ* 7.92 (d, *J* = 1.0 Hz, 1H), 7.64 (d, *J* = 7.9 Hz, 1H), 7.54 (dd, *J* = 7.8, 1.6 Hz, 1H), 7.40–7.36 (m, 1H), 7.21 (d, *J* = 0.6 Hz, 1H), 7.16 (d, *J* = 7.9 Hz, 1H), 6.88 (d, *J* = 0.5 Hz, 1H), 4.38 (s, 2H), 3.97 (d, *J* = 7.3 Hz, 2H), 3.20 (s, 3H), 2.72–2.64 (m, 1H), 2.60 (s, 3H), 2.00–1.76 (m, 4H), 1.68–1.59 (m, 2H); ^13^C NMR (CDCl_3_) *δ* 168.3, 156.3, 155.5, 141.4, 140.5, 138.3, 137.4, 133.1, 132.5, 127.9, 124.8, 124.3, 122.3 (2 ×), 119.5, 57.3, 51.9, 35.7, 29.5, 25.8, 24.3, 18.1; *m/z* MS (TOF ES^+^) 373.2; LC-MS *t*_R_: 2.25; HRMS - C_23_H_25_N_4_O [M+H]^+^ calcd 373.2028; found 373.2040.

#### 4.2.26 6-(3-(1-(Cyclohexylmethyl)-1H-pyrazol-4-yl)-6-methylpyridin-2-yl)-2-methylisoindolin-1-one (**6f**)

Synthesised according to General Procedure B. The crude product was purified by FCC (EtOAc: PE 2:8 → EtOAc 100% → DCM 100% → DCM: MeOH 9:1), followed by preparative HPLC (5–100%) to afford the title compound as a colourless oil (14 mg, 12%). ^1^H NMR (CDCl_3_) *δ* 7.93 (d, *J* = 0.9 Hz, 1H), 7.66 (d, *J* = 7.9 Hz, 1H), 7.53 (dd, *J* = 7.8, 1.5 Hz, 1H), 7.36 (d, *J* = 7.8 Hz, 1H), 7.26 (s, 1H), 7.17 (d, *J* = 7.9 Hz, 1H), 6.82 (s, 1H), 4.37 (s, 2H), 3.76 (d, *J* = 7.2 Hz, 2H), 3.19 (s, 3H), 2.61 (s, 3H), 1.76–1.59 (m, 4H), 1.49–1.42 (m, 2H), 1.25–1.04 (m, 3H), 0.85–0.74 (m, 2H); ^13^C NMR (CDCl_3_) *δ* 168.2, 156.3, 155.3, 141.0, 140.5, 138.3, 137.7, 133.1, 132.6, 128.7, 124.8, 124.5, 122.3, 122.4, 119.2, 58.6, 51.9, 38.6, 30.5, 29.5, 26.2, 25.6, 24.1; *m/z* MS (TOF ES^+^) 415.1 [M – H]^+^; LC-MS *t*_R_: 3.07; HRMS - C_25_H_29_N_4_O [M+H]^+^ calcd 401.2341; found 401.2350.

#### 4.2.27 2-Methyl-6-(6-methyl-3-(1-((tetrahydro-2H-pyran-4-yl)methyl)-1H-pyrazol-4-yl)pyridin-2-yl)isoindolin-1-one (**6g**)

Synthesised according to General Procedure B. The crude product was purified by FCC (EtOAc: PE 1:1 → EtOAc 100% → DCM → DCM: MeOH 9:1), followed by two rounds of purification via preparative HPLC (5–100%) to afford the title compound as a colourless oil (4 mg, 4%). Compound **7** was also isolated from this reaction, due to impure starting material. ^1^H NMR (CDCl_3_) *δ* 7.92–7.88 (m, 1H), 7.67 (d, *J* = 7.9 Hz, 1H), 7.62 (dd, *J* = 7.8, 1.6 Hz, 1H), 7.44–7.40 (m, 1H), 7.33 (d, *J* = 0.6 Hz, 1H), 7.20 (d, *J* = 7.9 Hz, 1H), 6.86 (d, *J* = 0.6 Hz, 1H), 4.41 (s, 2H), 3.96–3.90 (m, 2H), 3.84 (d, *J* = 7.3 Hz, 2H), 3.33 (td, *J* = 11.9, 2.1 Hz, 2H), 3.22 (s, 3H), 2.63 (s, 3H), 2.10–1.99 (m, 1H), 1.42–1.34 (m, 2H), 1.25–1.13 (m, 2H); ^13^C NMR (CDCl_3_) *δ* 168.2, 156.6, 155.6, 141.4, 140.4, 138.7, 137.5, 132.9, 132.5, 128.8, 122.5, 122.3, 124.8, 124.1, 119.6, 67.4, 57.9, 51.9, 36.1, 30.3, 29.5, 24.3; *m/z* MS (TOF ES^+^) hydrophobic 403.2; LC-MS *t*_R_: 2.52; HRMS - C_24_H_27_N_4_O_2_ [M+H]^+^ calcd 403.2134; found 403.2150.

2-Methyl-6-(6-methyl-2-(1-((tetrahydro-2*H*-pyran-4-yl)methyl)-1*H*-pyrazol-4-yl)pyridin-3-yl)isoindolin-1-one (**7**) was also isolated as a colourless oil (2 mg, 2%), due to impurities in the starting material. ^1^H NMR (CDCl_3_) *δ* 7.72–7.70 (m, 1H), 7.40–7.34 (m, 3H), 7.22 (d, *J* = 0.4 Hz, 1H), 7.10 (d, *J* = 0.6 Hz, 1H), 7.00 (d, *J* = 7.8 Hz, 1H), 4.36 (s, 2H), 3.89–3.82 (m, 2H), 3.77 (d, *J* = 7.2 Hz, 2H), 3.25 (td, *J* = 11.8, 2.1 Hz, 2H), 3.16 (s, 3H), 2.55 (s, 3H), 2.06–1.93 (m, 1H), 1.39–1.32 (m, 2H), 1.21–1.12 (m, 2H); ^13^C NMR (CDCl_3_) *δ* 168.2, 157.6, 149.0, 140.7, 140.2, 139.6, 138.5, 133.6, 132.6, 131.2, 130.3, 124.4, 122.7, 122.3, 120.6, 67.4, 57.8, 52.0, 36.0, 30.4, 30.0, 24.5; *m/z* MS (TOF ES^+^) hydrophobic 403.2; LC-MS *t*_R_: 2.51; HRMS - C_24_H_27_N_4_O_2_ [M+H]^+^ calcd 403.2134; found 403.2129.

#### 4.2.28 6-(3-(1-Benzyl-1H-pyrazol-4-yl)-6-methylpyridin-2-yl)-2-methylisoindolin-1-one (**6h**)

Synthesised according to General Procedure B. The crude product was purified by FCC (EtOAc: PE 3:7 → EtOAc 100% → DCM 100% → DCM: MeOH 9:1), followed by preparative HPLC (5–100%) to afford the title compound as a colourless oil (33 mg, 24%). ^1^H NMR (CDCl_3_) *δ* 7.88 (s, 1H), 7.63 (d, *J* = 7.9 Hz, 1H), 7.55–7.49 (m, 1H), 7.34–7.24 (m, 5H), 7.15 (d, *J* = 7.9 Hz, 1H), 7.10–7.05 (m, 2H), 6.90 (s, 1H), 5.16 (s, 2H), 4.34 (s, 2H), 3.20 (s, 3H), 2.59 (s, 3H); ^13^C NMR (CDCl_3_) *δ* 168.3, 156.5, 155.5, 141.1, 140.5, 138.8, 137.4, 136.2, 133.0, 132.5, 128.8, 128.4, 128.0, 127.6, 124.7, 124.2, 122.4, 122.3, 120.3, 56.0, 51.9, 29.5, 24.3; *m/z* MS (TOF ES^+^) 395.0; LC-MS *t*_R_: 3.78; HRMS - C_25_H_23_N_4_O [M+H]^+^ calcd 395.1872; found 395.1874.

#### 4.2.29 tert-Butyl 4-((4-(6-methyl-2-(2-methyl-3-oxoisoindolin-5-yl) pyridin-3-yl)-1H-pyrazol-1-yl)methyl)piperidine-1-carboxylate (**6i**)

Synthesised according to General Procedure B. The crude product was purified by FCC (EtOAc: PE 3:7 → EtOAc 100% → DCM 100% → DCM: MeOH 9:1), followed by preparative HPLC (5–100%) to afford the title compound as a colourless oil (25 mg, 20%). ^1^H NMR (CDCl_3_) *δ* 7.88 (s, 1H), 7.64 (d, *J* = 7.5 Hz, 1H), 7.54 (d, *J* = 7.4 Hz, 1H), 7.37 (d, *J* = 7.3 Hz, 1H), 7.29 (s, 1H), 7.17 (d, *J* = 7.5 Hz, 1H), 6.82 (s, 1H), 4.40 (s, 2H), 4.02 (d, *J* = 10.3 Hz, 2H), 3.80 (d, *J* = 6.5 Hz, 2H), 3.20 (s, 3H), 2.67–2.53 (m, 5H), 1.98–1.83 (m, 1H), 1.43 (s, 9H), 1.42–1.30 (m, 2H), 1.04–0.90 (m, 2H); ^13^C NMR (CDCl_3_) *δ* 168.3, 156.6, 155.6, 154.8, 141.2, 140.7, 138.8, 137.8, 133.1, 132.6, 129.0, 124.9, 124.3, 122.5 (2x), 119.6, 79.6, 57.7, 52.0, 42.9 (obtained from HSQC), 37.2, 29.6 (2 ×), 28.6, 24.3; *m/z* MS (TOF ES^+^) 502.1 [M – H]^+^; LC-MS *t*_R_: 3.96; HRMS - C_29_H_36_N_5_O_3_ [M+H]^+^ calcd 502.2818; found 502.2824.

#### 4.2.30 3-(1-(Cyclopentylmethyl)-1H-pyrazol-4-yl)-6-methyl-2-phenylpyridine (**6j**)

Synthesised according to General Procedure B. The crude product was purified by FCC (PE 100% → PE: EtOAc 1:1) followed by FCC (DCM 100% → DCM: MeOH 9:1) and preparative HPLC (30–100%) to afford the title compound as a colourless oil (6 mg, 5%). ^1^H NMR CDCl_3_) *δ* 7.66 (d, *J* = 7.9 Hz, 1H), 7.42–7.37 (m, 3H), 7.36–7.31 (m, 3H), 7.14 (d, *J* = 7.9 Hz, 1H), 6.75 (d, *J* = 0.6 Hz, 1H), 3.86 (d, *J* = 7.5 Hz, 2H), 2.61 (s, 3H), 2.33–2.19 (m, 1H), 1.62–1.49 (m, 6H), 1.16–1.05 (m, 2H); ^13^C NMR (CDCl_3_) *δ* 156.4, 156.1, 141.1, 138.1, 137.0, 129.3, 128.3 (2 ×), 127.8, 124.2, 122.0, 119.6, 57.0, 40.7, 30.1, 24.9, 24.3; *m/z* MS (TOF ES^+^) 318.2 [M – H]^+^; LC-MS *t*_R_: 2.51; HRMS - C_21_H_24_N_3_ [M+H]^+^ calcd 318.1970; found 318.1970.

#### 4.2.31 3-(1-(Cyclopentylmethyl)-1H-pyrazol-4-yl)-2-(3,4-difluorophenyl)-6-methylpyridine (**6k**)

Synthesised according to General Procedure B. The crude product was purified by FCC (PE: EtOAc 8:2 → EtOAc 100%), followed by pre-parative HPLC (30–100%) to afford the title compound as a colourless oil (10 mg, 16%). ^1^H NMR (CDCl_3_) *δ* 7.67 (d, *J* = 7.9 Hz, 1H), 7.37 (s, 1H), 7.32–7.25 (m, 1H), 7.21–7.09 (m, 3H), 6.93 (s, 1H), 3.95 (d, *J* = 7.6 Hz, 2H), 2.63 (s, 3H), 2.40–2.29 (m, 1H), 1.71–1.53 (m, 6H), 1.23–1.11 (m, 2H); ^13^C NMR (CDCl_3_) *δ* 156.4, 153.9, 150.3 (dd, *J*_*C,F*_ = 250.5, 23.2 Hz), 150.2 (dd, *J*_*C,F*_ = 249.5, 23.2 Hz), 138.2, 137.9, 137.7 (dd, *J* = 5.8, 3.5 Hz), 128.2, 125.8 (dd, *J*_*C,F*_ = 6.3, 3.6 Hz), 124.4, 122.6, 119.2, 118.7 (d, *J*_*C,F*_ = 17.8 Hz), 117.1 (d, *J*_*C,F*_ = 17.3 Hz), 57.1, 40.7, 30.1, 24.9, 24.2; *m/z* MS (TOF ES^+^) 354.0 [M – H]^+^; LC-MS *t*_R_: 3.73; HRMS - C_21_H_22_F_2_N_3_ [M+H]^+^ calcd 354.1782; found 354.1784.

#### 4.2.32 4-(3-(1-(Cyclopentylmethyl)-1H-pyrazol-4-yl)-6-methylpyridin-2-yl)-2-nitroaniline (**6l**)

Synthesised according to General Procedure B. The crude product was purified by FCC (PE: EtOAc 8:2 → EtOAc 100%), followed by FCC (DCM 100% → DCM: MeOH 9:1) to afford the title compound as a yellow oil (16 mg, 23%). ^1^H NMR (CDCl_3_) *δ* 8.30 (d, *J* = 2.0 Hz, 1H), 7.60 (d, *J* = 7.9 Hz, 1H), 7.41 (dd, *J* = 8.6, 2.1 Hz, 1H), 7.33 (d, *J* = 0.6 Hz, 1H), 7.13 (d, *J* = 7.9 Hz, 1H), 7.08 (d, *J* = 0.6 Hz, 1H), 6.72 (d, *J* = 8.6 Hz, 1H), 6.17 (br s, 2H), 3.94 (d, *J* = 7.6 Hz, 2H), 2.60 (s, 3H), 2.39–2.28 (m, 1H), 1.66–1.53 (m, 6H), 1.20–1.11 (m, 2H); ^13^C NMR (CDCl_3_) *δ* 156.6, 153.9, 144.3, 138.3, 138.0, 136.9, 132.0, 129.9, 128.2, 127.3, 124.1, 122.1, 119.5, 118.3, 57.1, 40.8, 30.1, 25.0, 24.2; *m/z* MS (TOF ES^+^) 378.0 [M – H]^+^; LC-MS *t*_R_: 3.43; HRMS - C_21_H_24_N_5_O_2_ [M+H]^+^ calcd 378.1930; found 378.1932.

#### 4.2.33 4-(3-(1-(Cyclopentylmethyl)-1H-pyrazol-4-yl)-6-methylpyridin-2-yl)aniline (**6m**)

Synthesised according to General Procedure B. The crude product was purified by (PE 100% → EtOAc 100%), followed by preparative HPLC (5–100%) to afford the title compound as a light-yellow oil (187 mg, 82%). ^1^H NMR (CDCl_3_) *δ* 7.59 (d, *J* = 7.9 Hz, 1H), 7.38 (d, *J* = 0.6 Hz, 1H), 7.22–7.18 (m, 2H), 7.05 (d, *J* = 7.9 Hz, 1H), 6.90 (d, *J* = 0.5 Hz, 1H), 6.63–6.58 (m, 2H), 3.89 (d, *J* = 7.5 Hz, 2H), 3.56 (br s, 2H), 2.57 (s, 3H), 2.36–2.25 (m, 1H), 1.67–1.49 (m, 6H), 1.20–1.09 (m, 2H); ^13^C NMR (CDCl_3_) *δ* 156.4, 155.9, 146.4, 138.1, 137.2, 131.1, 130.5, 128.3, 124.0, 121.3, 120.0, 114.7, 57.0, 40.7, 30.1, 25.0, 24.3; *m/z* MS (TOF ES^+^) 333.2; LC-MS *t*_R_: 2.41; HRMS - C_21_H_25_N_4_ [M+H]^+^ calcd 333.2079; found 333.2080.

#### 4.2.34 3-(3-(1-(Cyclopentylmethyl)-1H-pyrazol-4-yl)-6-methylpyridin-2-yl)aniline (**6n**)

Synthesised according to General Procedure B. The crude product was purified by (EtOAc: PE 1:1 → EtOAc 100%) and (DCM 100% → DCM: MeOH 9:1), followed by preparative HPLC (5–100%) to afford the title compound as a light-yellow oil (135 mg, 69%). ^1^H NMR (CDCl_3_) *δ* 7.58 (d, *J* = 7.9 Hz, 1H), 7.38–7.35 (m, 1H), 7.04 (d, *J* = 8.0 Hz, 1H), 7.00 (t, *J* = 7.8 Hz, 1H), 6.77 (s, 1H), 6.68–6.65 (m, 1H), 6.65–6.61 (m, 1H), 6.58–6.54 (m, 1H), 3.80 (d, *J* = 7.6 Hz, 2H), 3.60 (s, 2H), 2.52 (s, 3H), 2.27–2.17 (m, 1H), 1.59–1.41 (m, 6H), 1.11–1.00 (m, 2H); ^13^C NMR (CDCl_3_) *δ* 156.3, 155.5, 146.6, 142.0, 137.7, 136.5, 128.9, 128.3, 124.0, 121.8, 119.3 (2 ×), 115.7, 114.5, 56.7, 40.5, 29.9, 24.8, 24.1; *m/z* MS (TOF ES^+^) 333.2; LC-MS *t*_R_: 2.75; HRMS - C_21_H_25_N_4_ [M+H]^+^ calcd 333.2079; found 333.2081.

#### 4.2.35 3-(3-(1-(Cyclopentylmethyl)-1H-pyrazol-4-yl)-6-methylpyridin-2-yl)-N,N-dimethylbenzamide (**6o**)

Synthesised according to General Procedure B. The crude product was purified by FCC (PE: EtOAc 8:2 → EtOAc 100% → EtOAc: MeOH 9:1), followed by FCC (DCM 100% → DCM: MeOH 9:1) to afford the title compound as a colourless oil (25 mg, 35%). ^1^H NMR (CDCl_3_) *δ* 7.58 (d, *J* = 7.9 Hz, 1H), 7.48–7.44 (m, 1H), 7.39 (dt, *J* = 7.5, 1.6 Hz, 1H), 7.35 (dt, *J* = 7.7, 1.5 Hz, 1H), 7.31–7.26 (m, 2H), 7.09 (d, *J* = 8.0 Hz, 1H), 6.81 (d, *J* = 0.5 Hz, 1H), 3.81 (d, *J* = 7.6 Hz, 2H), 3.00 (s, 3H), 2.87 (s, 3H), 2.54 (s, 3H), 2.28–2.16 (m, 1H), 1.59–1.42 (m, 6H), 1.12–1.02 (m, 2H); ^13^C NMR (CDCl_3_) *δ* 171.2, 156.3, 155.2, 140.9, 138.1, 137.6, 136.3, 130.7, 128.3, 128.2, 128.1, 126.9, 124.4, 122.3, 119.4, 57.0, 40.7, 39.6, 35.4, 30.1, 24.9, 24.2; *m/z* MS (TOF ES^+^) (hydrophobic) 389.1 [M – H]^+^; LC-MS *t*_R_: 2.47; HRMS - C_24_H_29_N_4_O [M+H]^+^ calcd 389.2341; found 389.2343.

#### 4.2.36 3-(3-(1-(Cyclopentylmethyl)-1H-pyrazol-4-yl)-6-methylpyridin-2-yl)-N-methylbenzamide (**6p**)

Synthesised according to General Procedure B. The crude product was purified by FCC (PE: EtOAc 8:2 → EtOAc 100%), followed by FCC (DCM 100% → DCM: MeOH 9:1) to afford the title compound as a colourless oil (19 mg, 28%). ^1^H NMR (CDCl_3_) *δ* 7.84–7.80 (m, 2H), 7.70–7.67 (m, 1H), 7.50–7.47 (m, 1H), 7.40–7.34 (m, 2H), 7.18 (d, *J* = 7.9 Hz, 1H), 6.78 (s, 1H), 6.46 (br s, 1H), 3.87 (d, 2H), 3.01–2.95 (m, 3H), 2.61 (s, 3H), 2.31–2.24 (m, 1H), 1.65–1.50 (m, 6H), 1.17–1.05 (m, 2H); ^13^C NMR (CDCl_3_) *δ* 168.0, 156.3, 155.1, 140.9, 138.1, 137.5, 134.9, 132.3, 128.5, 128.3, 127.5, 127.1, 124.4, 122.4, 119.2, 57.0, 40.7, 30.1, 26.8, 24.9, 24.1; *m/z* MS (TOF ES^+^) 375.0 [M – H]^+^; LC-MS *t*_R_: 3.18; HRMS - C_23_H_27_N_4_O [M+H]^+^ calcd 375.2185; found 375.2185.

#### 4.2.37 3-(3-(1-(Cyclopentylmethyl)-1H-pyrazol-4-yl)-6-methylpyridin-2-yl)benzamide (**6q**)

Synthesised according to General Procedure B. The crude product was purified by FCC (PE: EtOAc 8:2 → EtOAc 100%), followed by FCC (DCM 100% → DCM: MeOH 9:1) to afford the title compound as a colourless oil (15 mg, 23%). ^1^H NMR (CDCl_3_) *δ* 7.89 (t, *J* = 1.6 Hz, 1H), 7.87–7.82 (m, 1H), 7.67 (d, *J* = 7.9 Hz, 1H), 7.52 (dt, *J* = 7.6, 1.3 Hz, 1H), 7.41–7.34 (m, 2H), 7.17 (d, *J* = 7.9 Hz, 1H), 6.79 (s, 1H), 6.29 (br s, 1H), 5.82 (br s, 1H), 3.86 (d, *J* = 7.6 Hz, 2H), 2.61 (s, 3H), 2.34–2.20 (m, 1H), 1.64–1.48 (m, 6H), 1.14–1.05 (m, 2H); ^13^C NMR (CDCl_3_) *δ* 169.2, 156.4, 155.0, 141.1, 138.1, 137.6, 133.7, 133.0, 128.5, 128.3, 128.2, 127.4, 124.5, 122.5, 119.2, 57.1, 40.7, 30.1, 24.9, 24.2; *m/z* MS (TOF ES^+^) 361.0 [M – H]^+^; LC-MS *t*_R_: 3.12; HRMS - C_22_H_25_N_4_O [M+H]^+^ calcd 361.2028; found 361.2027.

#### 4.2.38 N-(3-(3-(1-(Cyclopentylmethyl)-1H-pyrazol-4-yl)-6-methylpyridin-2-yl)phenyl)acetamide (**6r**)

Synthesised according to General Procedure B. The crude product was purified by FCC (PE: EtOAc 8:2 → EtOAc 100%), followed by FCC (DCM 100% → DCM: MeOH 9:1) to afford the title compound as a colourless oil (14 mg, 21%). ^1^H NMR (CDCl_3_) *δ* 7.76 (s, 1H), 7.67–7.60 (m, 2H), 7.35 (s, 2H), 7.17 (t, *J* = 7.9 Hz, 1H), 7.09 (d, *J* = 8.0 Hz, 1H), 7.02–6.95 (m, 1H), 6.76 (s, 1H), 3.80 (d, *J* = 7.6 Hz, 2H), 2.53 (s, 3H), 2.27–2.17 (m, 1H), 2.00 (s, 3H), 1.58–1.40 (m, 6H), 1.10–0.98 (m, 2H); ^13^C NMR (CDCl_3_) *δ* 168.3, 155.8, 155.6, 141.2, 138.4, 138.1, 137.4, 128.9, 128.5, 124.9, 124.6, 122.4, 120.4, 119.5, 119.1, 57.0, 40.7, 30.1, 24.9, 24.5, 24.0; *m/z* MS (TOF ES^+^) 375.0 [M – H]^+^; LC-MS *t*_R_: 3.11; HRMS - C_23_H_27_N_4_O [M+H]^+^ calcd 375.2185; found 375.2185.

#### 4.2.39 6-(3-(1-(Cyclopentylmethyl)-1H-pyrazol-4-yl)-6-methylpyridin-2-yl)isoindolin-1-one (**6s**)

Synthesised according to General Procedure B. The crude product was purified by FCC (EtOAc: PE 2:8 → EtOAc 100% → DCM 100% →DCM: MeOH 9:1) to afford the title compound as a dark purple oil (112 mg, 21%). ^1^H NMR (CDCl_3_) *δ* 7.95 (d, *J* = 0.8 Hz, 1H), 7.72 (br s, 1H), 7.66 (d, *J* = 7.9 Hz, 1H), 7.60 (dd, *J* = 7.8, 1.5 Hz, 1H), 7.42 (d, *J* = 7.8 Hz, 1H), 7.27 (d, *J* = 0.5 Hz, 1H), 7.17 (d, *J* = 7.9 Hz, 1H), 6.87 (s, 1H), 4.45 (s, 2H), 3.86 (d, *J* = 7.5 Hz, 2H), 2.60 (s, 3H), 2.30–2.23 (m, 1H), 1.62–1.45 (m, 6H), 1.13–1.01 (m, 2H); ^13^C NMR (CDCl_3_) *δ* 171.7, 156.4, 155.3, 143.2, 141.2, 138.2, 137.6, 133.2, 132.3, 128.3, 124.9, 124.5, 123.0, 122.4, 119.4, 57.0, 45.6, 40.7, 30.1, 24.9, 24.2; *m/z* MS (TOF ES^+^) 373.0 [M – H]^+^; LC-MS *t*_R_: 3.03; HRMS - C_23_H_25_N_4_O [M+H]^+^ calcd 373.2028; found 373.2027.

#### 4.2.40 6-(3-(1-(Cyclopentylmethyl)-1H-pyrazol-4-yl)-6-methylpyridin-2-yl)-2-propylisoindolin-1-one (**6t**)

Synthesised according to General Procedure B. The crude product was purified by FCC (EtOAc: PE 2:8 → EtOAc 100%), followed by preparative HPLC (5–100%) to afford the title compound as a colourless oil (12 mg, 20%). ^1^H NMR (CDCl_3_) *δ* 7.93 (d, *J* = 0.9 Hz, 1H), 7.67 (d, *J* = 7.9 Hz, 1H), 7.54 (dd, *J* = 7.8, 1.5 Hz, 1H), 7.39 (d, *J* = 7.8 Hz, 1H), 7.25 (s, 1H), 7.18 (d, *J* = 8.0 Hz, 1H), 6.89 (s, 1H), 4.38 (s, 2H), 3.87 (d, *J* = 7.5 Hz, 2H), 3.61–3.54 (m, 2H), 2.62 (s, 3H), 2.30–2.22 (m, 1H), 1.75–1.64 (m, 2H), 1.62–1.45 (m, 6H), 1.14–1.03 (m, 2H), 0.96 (t, *J* = 7.4 Hz, 3H); ^13^C NMR (CDCl_3_) *δ* 168.1, 156.2, 155.3, 140.9, 140.8, 138.2, 137.7, 133.3, 132.5, 128.3, 124.8, 124.6, 122.5 (2 ×), 119.3, 57.0, 49.8, 44.1, 40.6, 30.1, 24.9, 24.0, 21.7, 11.3; *m/z* MS (TOF ES^+^) 415.1 [M – H]^+^; LC-MS *t*_R_: 3.07; HRMS - C_26_H_31_N_4_O [M+H]^+^ calcd 415.2498; found 415.2505.

#### 4.2.41 6-(3-(1-(Cyclopentylmethyl)-1H-pyrazol-4-yl)-6-methylpyridin-2-yl)indolin-2-one (**6u**)

Synthesised according to General Procedure B. The crude product was purified by column chromatography (EtOAc: PE 1:1 → EtOAc 100%), followed by preparative HPLC (5–100%) to afford the title compound as a light-purple oil (18 mg, 22%). ^1^H NMR (CDCl_3_) *δ* 8.06 (s, 1H), 7.65 (d, *J* = 7.9 Hz, 1H), 7.35 (d, *J* = 0.5 Hz, 1H), 7.15 (d, *J* = 7.9 Hz, 2H), 7.03 (dd, *J* = 7.6, 1.4 Hz, 1H), 6.94 (d, *J* = 1.0 Hz, 1H), 6.92–6.89 (m, 1H), 3.90 (d, *J* = 7.6 Hz, 2H), 3.52 (s, 2H), 2.60 (s, 3H), 2.35–2.25 (m, 1H), 1.65–1.47 (m, 6H), 1.16–1.06 (m, 2H); ^13^C NMR (CDCl_3_) *δ* 176.9, 156.2, 155.8, 142.4, 141.2, 138.2, 137.3, 128.2, 124.9, 124.3, 124.2, 123.7, 122.2, 119.5, 110.6, 57.0, 40.7, 36.0, 30.1, 24.9, 24.3; *m/z* MS (TOF ES^+^) hydrophobic 373.2; LC-MS *t*_R_: 2.92; HRMS - C_23_H_25_N_4_O [M+H]^+^ calcd 373.2028; found 373.2038.

#### 4.2.42 tert-Butyl 4-(2-chloropyridin-3-yl)-1H-pyrazole-1-carboxylate (**9a**)

Synthesised according to General Procedure C starting from 3-bromo-2-chloropyridine. The crude product was absorbed on silica gel and purified by FCC (PE: EtOAc 1:1 → EtOAc 100%) to afford the title compound as a white/colourless resin (400 mg, 28%). ^1^H NMR (CDCl_3_) *δ* 8.42 (d, *J* = 0.8 Hz, 1H), 8.28 (dd, *J* = 4.7, 1.9 Hz, 1H), 7.97 (d, *J* = 0.7 Hz, 1H), 7.73 (dd, *J* = 7.7, 1.9 Hz, 1H), 7.23 (dd, *J* = 7.7, 4.7 Hz, 1H), 1.61 (s, 9H); *m/z* MS (TOF ES^+^) 180.0 [M-Boc group]^+^; LC-MS *t*_R_: 4.15.

#### 4.2.43 2-Chloro-3-(1h-pyrazol-4-yl)pyridine (**10a**)

*tert*-Butyl 4-(2-chloropyridin-3-yl)-1*H*-pyrazole-1-carboxylate (400 mg, 1.43 mmol, 1.00 equiv.) was dissolved in DCM (15 mL) and TFA (2 mL) was dropwise added. The reaction was stirred at room temperature for 6 h, before more DCM (100 mL) was added and the organic solution was washed with 1 M NaOH (100 mL), water (100 mL) and brine (100 mL). The organic layer was dried with Na_2_SO_4,_ filtered and evaporated to dryness under reduced pressure. The residue was purified by FCC (DCM 100% → DCM: MeOH 9:1) to afford the title compound as white solid (119 mg, 46%). ^1^H NMR (CDCl_3_) *δ* 8.31 (dd, *J* = 4.7, 1.8 Hz, 1H), 8.06 (br s, 2H), 7.84–7.77 (m, 1H), 7.28 (dd, *J* = 7.7, 4.7 Hz, 1H); *m/z* MS (TOF ES^+^) 180.0 [M – H]^+^; LC-MS *t*_R_: 3.92.

#### 4.2.44 2-Chloro-3-(1-(cyclopentylmethyl)-1H-pyrazol-4-yl)pyridine (**11a**)

Synthesised according to General Procedure A. The crude product was purified by FCC (PE 100%→ EtOAc 100%) to afford the title compound as a colourless oil (97 mg, 55%). ^1^H NMR (CDCl_3_) *δ* 8.27 (dd, *J* = 4.7, 1.9 Hz, 1H), 7.90 (s, 1H), 7.83–7.77 (m, 2H), 7.28–7.23 (m, 1H), 4.10 (d, *J* = 7.5 Hz, 2H), 2.55–2.42 (m, 1H), 1.81–1.54 (m, 6H), 1.35–1.24 (m, 2H); *m/z* MS (hydrophobic method) (TOF ES^+^) 262.0 [M – H]^+^; LC-MS *t*_R_: 3.45.

#### 4.2.45 4-(2-Bromophenyl)-1-(cyclopentylmethyl)-1H-pyrazole (**11b**)

Synthesised according to General Procedure B starting from 1-bromo-2-iodobenzene. The crude product was purified by FCC (PE 100% → PE: EtOAc 1:1), followed by FCC (DCM 100%) to afford the title compound as a colourless oil (100 mg, 31%). ^1^H NMR (CDCl_3_) *δ* 7.71–7.68 (m, 1H), 7.68–7.64 (m, 1H), 7.54 (dd, *J* = 8.0, 1.2 Hz, 1H), 7.32 (dd, *J* = 7.7, 1.7 Hz, 1H), 7.20 (td, *J* = 7.5, 1.3 Hz, 1H), 7.00 (ddd, *J* = 7.9, 7.5, 1.7 Hz, 1H), 3.98 (d, *J* = 6.5 Hz, 2H), 2.44–2.32 (m, 1H), 1.70–1.44 (m, 6H), 1.25–1.14 (m, 2H); *m/z* MS (TOF ES^+^) 305.0; LC-MS *t*_R_: 3.95.

#### 4.2.46 3-Bromo-4-(1-(cyclopentylmethyl)-1H-pyrazol-4-yl)pyridine (**11c**)

Synthesised according to General Procedure B starting from 3-bromo-4-iodopyridine. The crude product was purified by FCC (PE 100% → PE: EtOAc 30:70), followed by FCC (DCM 100% → DCM: MeOH 9:1) to afford the title compound as a colourless oil (51 mg, 16%). ^1^H NMR (CDCl_3_) *δ* 8.71 (s, 1H), 8.41 (d, *J* = 5.1 Hz, 1H), 8.04–7.99 (m, 1H), 7.90–7.85 (m, 1H), 7.33 (d, *J* = 5.1 Hz, 1H), 4.08 (d, *J* = 7.5 Hz, 2H), 2.51–2.39 (m, 1H), 1.79–1.50 (m, 6H), 1.32–1.22 (m, 2H); *m/z* MS (TOF ES^+^) 306.1; LC-MS *t*_R_: 3.49.

#### 4.2.47 4-Bromo-3-(1-(cyclopentylmethyl)-1H-pyrazol-4-yl)pyridine (**11d**)

Synthesised according to General Procedure B starting from 4-bromo-3-iodopyridine. The crude product was purified by FCC (PE 100% → PE: EtOAc 30:70) to afford the title compound as a colourless oil (95 mg, 29%). ^1^H NMR (CDCl_3_) *δ* 8.60 (s, 1H), 8.24 (d, *J* = 5.3 Hz, 1H), 7.84–7.77 (m, 2H), 7.57 (d, *J* = 5.4 Hz, 1H), 4.10 (d, *J* = 7.5 Hz, 2H), 2.55–2.42 (m, 1H), 1.80–1.54 (m, 6H), 1.35–1.25 (m, 2H), *m/z* MS (TOF ES^+^) 306.1; LC-MS *t*_R_: 3.56.

#### 4.2.48 2-Bromo-3-(1-(cyclopentylmethyl)-1H-pyrazol-4-yl)pyrazine (**11e**)

Synthesised according to General Procedure C starting from 2,3-dibromopyrazine. The crude product was purified by FCC (PE 100% → EtOAc 100%) to afford the title compound as a colourless oil (44 mg, 8%). ^1^H NMR (CDCl_3_) *δ* 8.40 (d, *J* = 2.3 Hz, 1H), 8.27–8.16 (m, 2H), 8.07 (d, *J* = 2.3 Hz, 1H), 4.04 (d, *J* = 7.5 Hz, 2H), 2.50–2.33 (m, 1H), 1.74–1.46 (m, 6H), 1.31–1.16 (m, 2H); *m/z* MS (TOF ES^+^) 370.0 [M – H]^+^; LC-MS *t*_R_: 3.54.

#### 4.2.49 6-(3-(1-(Cyclopentylmethyl)-1H-pyrazol-4-yl)pyridin-2-yl)-2-methylisoindolin-1-one (**12a**)

Synthesised according to General Procedure C. The crude product was purified by FCC (PE 100% → EtOAc 100% → DCM 100% → DCM: MeOH 9:1), followed by preparative HPLC (5–100%) to afford the title compound as a colourless oil (5 mg, 7%). ^1^H NMR (CDCl_3_) *δ* 8.59 (dd, *J* = 4.8, 1.6 Hz, 1H), 7.90 (d, *J* = 1.0 Hz, 1H), 7.79 (dd, *J* = 7.8, 1.6 Hz, 1H), 7.60 (dd, *J* = 7.8, 1.6 Hz, 1H), 7.42 (d, *J* = 7.8 Hz, 1H), 7.32 (dd, *J* = 7.8, 4.8 Hz, 1H), 7.28 (d, *J* = 0.5 Hz, 1H), 6.93–6.91 (m, 1H), 4.40 (s, 2H), 3.88 (d, *J* = 7.6 Hz, 2H), 3.20 (s, 3H), 2.33–2.22 (m, 1H), 1.64–1.45 (m, 6H), 1.13–1.02 (m, 2H); ^13^C NMR (CDCl_3_) *δ* 168.3, 156.2, 147.4, 141.0, 140.8, 138.4, 137.4, 133.2, 132.6, 128.5, 127.8, 124.8, 122.9, 122.6, 119.4, 57.2, 52.0, 40.8, 30.3, 29.6, 25.1; *m/z* MS (TOF ES^+^) 373.0 [M – H]^+^; LC-MS *t*_R_: 4.18; HRMS - C_23_H_25_N_4_O [M+H]^+^ calcd 373.2028; found 373.2033.

#### 4.2.50 6-(2-(1-(Cyclopentylmethyl)-1H-pyrazol-4-yl)phenyl)-2-methylisoindolin-1-one (**12b**)

Synthesised according to General Procedure B. The crude product was purified by FCC (PE 100% → EtOAc 100%), followed by prep HPLC (5–100%) to afford the title compound as a colourless oil (22 mg, 39%). ^1^H NMR (CDCl_3_) *δ* 7.79 (t, *J* = 1.0 Hz, 1H), 7.48–7.44 (m, 1H), 7.40–7.30 (m, 5H), 7.23 (d, *J* = 0.6 Hz, 1H), 6.82 (s, 1H), 4.38 (s, 2H), 3.85 (d, *J* = Hz, 2H), 3.21 (s, 3H), 2.32–2.21 (m, 1H), 1.62–1.46 (m, 6H), 1.12–1.02 (m, 2H); ^13^C NMR (CDCl_3_) *δ* 168.5, 142.4, 139.6, 139.4, 138.3, 133.2, 133.0, 131.4, 130.6, 129.3, 128.4, 128.0, 126.8, 124.4, 122.2, 121.3, 56.9, 51.9, 40.7, 30.1, 29.5, 24.9; *m/z* MS (TOF ES^+^) hydrophobic 372.2; LC-MS *t*_R_: 3.59; HRMS - C_24_H_26_N_3_O [M+H]^+^ calcd 372.2076; found 372.2075.

#### 4.2.51 6-(4-(1-(Cyclopentylmethyl)-1H-pyrazol-4-yl)pyridin-3-yl)-2-methylisoindolin-1-one (**12c**)

Synthesised according to General Procedure B. The crude product was purified by FCC (EtOAc: PE 1:1 → EtOAc 100% → DCM → DCM: MeOH 9:1), followed by preparative HPLC (5–100%) to afford the title compound as a colourless oil (27 mg, 44%). ^1^H NMR (CDCl_3_) *δ* 8.58 (d, *J* = 4.8 Hz, 1H), 8.50 (s, 1H), 7.82 (d, *J* = 0.7 Hz, 1H), 7.49–7.39 (m, 3H), 7.31 (d, *J* = 0.5 Hz, 1H), 6.95 (d, *J* = 0.4 Hz, 1H), 4.45 (s, 2H), 3.89 (d, *J* = 7.5 Hz, 2H), 3.25 (s, 3H), 2.34–2.24 (m, 1H), 1.66–1.50 (m, 6H), 1.17–1.04 (m, 2H); ^13^C NMR (CDCl_3_) *δ* 168.1, 163.5, 150.9, 149.1, 140.5, 139.2, 138.8, 138.5, 133.6, 132.8, 128.9, 124.6, 122.8, 122.4, 118.7, 57.1, 51.9, 40.6, 30.1, 29.6, 24.9; *m/z* MS (TOF ES^+^) hydro-phobic 372.2; LC-MS *t*_R_: 3.11; HRMS - C_23_H_25_N_4_O [M+H]^+^ calcd 373.2028; found 373.2025.

#### 4.2.52 6-(3-(1-(Cyclopentylmethyl)-1H-pyrazol-4-yl)pyridin-4-yl)-2-methylisoindolin-1-one (**12d**)

Synthesised according to General Procedure B. The crude product was purified by FCC (EtOAc: PE 1:1 → EtOAc 100% → DCM → DCM: MeOH 9:1), followed by preparative HPLC (5–100%) to afford the title compound as a colourless oil (38 mg, 63%). ^1^H NMR (CDCl_3_) *δ* 8.72 (s, 1H), 8.55 (d, *J* = 5.0 Hz, 1H), 7.80 (d, *J* = 0.6 Hz, 1H), 7.43–7.39 (m, 1H), 7.37 (dd, *J* = 7.8, 1.5 Hz, 1H), 7.25 (d, *J* = 5.0 Hz, 1H), 7.23 (d, *J* = 0.6 Hz, 1H), 7.04–6.99 (m, 1H), 4.43 (s, 2H), 3.92 (d, *J* = 7.5 Hz, 2H), 3.24 (s, 3H), 2.37–2.27 (m, 1H), 1.67–1.50 (m, 6H), 1.18–1.07 (m, 2H); ^13^C NMR (CDCl_3_) *δ* 168.1, 150.9, 149.1, 140.5, 139.2, 138.8, 138.5, 134.1, 133.6, 132.8, 128.9, 124.6, 122.8, 122.4, 118.7, 57.1, 51.9, 40.6, 30.1, 29.6, 24.9; *m/z* MS (TOF ES^+^) hydrophobic 373.2; LC-MS *t*_R_: 3.17; HRMS - C_23_H_25_N_4_O [M+H]^+^ calcd 373.2028; found 373.2028.

#### 4.2.53 6-(3-(1-(Cyclopentylmethyl)-1H-pyrazol-4-yl)pyrazin-2-yl)-2-methylisoindolin-1-one (**12e**)

Synthesised according to General Procedure B. The crude product was purified by (EtOAc: PE 1:1 → EtOAc 100% → DCM→ DCM: MeOH 9:1), followed by preparative HPLC (5–100%) to afford the title compound as a colourless oil (16 mg, 35%). ^1^H NMR (CDCl_3_) *δ* 8.51 (d, *J* = 2.4 Hz, 1H), 8.44 (d, *J* = 2.4 Hz, 1H), 7.99 (d, *J* = 1.0 Hz, 1H), 7.67 (dd, *J* = 7.8, 1.6 Hz, 1H), 7.51 (dd, *J* = 7.8, 0.6 Hz, 1H), 7.41 (s, 1H), 7.23 (d, *J* = 0.5 Hz, 1H), 4.44 (s, 2H), 3.91 (d, *J* = 7.5 Hz, 2H), 3.22 (s, 3H), 2.39–2.28 (m, 1H), 1.69–1.47 (m, 6H), 1.20–1.10 (m, 2H); ^13^C NMR (CDCl_3_) *δ* 167.9, 151.0, 146.3, 142.7, 141.5, 140.8, 139.5, 139.1, 133.6, 132.1, 129.9, 124.4, 123.0, 120.1, 57.2, 52.0, 40.5, 30.2, 29.6, 24.9; *m/z* MS (TOF ES^+^) 374.2; LC-MS *t*_R_: 2.76; HRMS - C_22_H_24_N_5_O [M+H]^+^ calcd 374.1981; found 374.1980.

#### 4.2.54 2-Chloro-6-methyl-3-((trimethylsilyl)ethynyl)pyridine (**13**)

3-Bromo-2-chloro-6-methylpyridine (1.00 g, 4.84 mmol, 1.00 equiv.), ethynyltrimethylsilane (951 mg, 9.69 mmol, 2.00 equiv.), Pd (PPh_3_)_2_Cl_2_ (170 mg, 242 μmol, 0.05 equiv.)and CuI (92.2 mg, 0.484 μmol, 0.10 equiv.) were suspended in DIPEA (10 mL) and the reaction mixture was heated up to 100 °C in a sealed microwave tube for 17 h. EtOAc (100 mL) was added to the reaction mixture and the organic layer was washed with water (2 × 100 mL) and brine (100 mL). The organic layer was dried with Na_2_SO_4,_ filtered and evaporated to dryness under reduced pressure. The crude product was purified by FCC (EtOAc: PE 1:9 → EtOAc: PE 1:1) to afford the title compound as a beige solid (320 mg, 30%). ^1^H NMR (CDCl_3_) *δ* 7.65 (d, *J* = 7.8 Hz, 1H), 7.02 (d, *J* = 7.8 Hz, 1H), 2.52 (s, 3H), 0.26 (s, 9H); *m/z* MS (TOF ES^+^) 224.0; LC-MS *t*_R_: 2.28.

#### 4.2.55 2-Chloro-3-ethynyl-6-methylpyridine (**14**)

Potassium carbonate (70 mg, 501 μmol, 0.35 equiv.) was added to a solution of 2-chloro-6-methyl-3-((trimethylsilyl)ethynyl)pyridine (320 mg, 1.43 mmol, 1.0 equiv.) in MeOH (10 mL) and the reaction mixture was stirred at room temperature for 1 h. The solvent was removed under reduced pressure and the residue was taken up in DCM (100 mL) and washed with water (2 × 100 mL) and brine (100 mL). The organic layer was dried with Na_2_SO_4,_ filtered and evaporated to dryness under reduced pressure. The title compound was obtained as a brown solid (203 mg, 94%). The product was used in the next step without further purification. ^1^H NMR (CDCl_3_) *δ* 7.68 (d, *J* = 7.8 Hz, 1H), 7.04 (d, *J* = 7.8 Hz, 1H), 3.41 (s, 1H), 2.52 (s, 3H); *m/z* MS (TOF ES^+^) no ionization; LC-MS *t*_R_: 2.76.

#### 4.2.56 2-Chloro-3-(1-(cyclopentylmethyl)-1H-1,2,3-triazol-4-yl)-6-methylpyridine (**15**)

2-Chloro-3-ethynyl-6-methylpyridine (145 mg, 957 μmol, 1.0 equiv) and (azidomethyl)cyclopentane (1 M solution in 2-methoxy-2methyl-propane) (957 μL, 957 μmol, 1.0 equiv) were dissolved in a mixture of *t*-BuOH and water (6 mL, 1:1 ratio). Copper (II) sulfate pentahydrate (47.8 mg, 191 μmol, 0.2 equiv), followed by sodium ascorbate (75.8 mg, 383 μmol, 0.4 equiv) were added and the reaction mixture was stirred at room temperature for 17 h. Then EtOAc (100 mL) was added and mixture was washed with water (2 × 100 mL) and brine (100 mL). The organic layer was dried with Na_2_SO_4,_ filtered and evaporated to dryness under reduced pressure to afford the title compound as a brown solid (240 mg, 91%). The product was used in the next step without further purification. ^1^H NMR (CDCl_3_) *δ* 8.50 (d, *J* = 7.9 Hz, 1H), 8.19 (s, 1H), 7.21 (d, *J* = 7.8 Hz, 1H), 4.35 (d, *J* = 7.6 Hz, 2H), 2.56 (s, 3H), 2.54–2.45 (m, 1H), 1.82–1.57 (m, 6H), 1.37–1.27 (m, 2H); *m/z* MS (TOF ES^+^) 277.1; LC-MS *t*_R_: 3.03.

#### 4.2.57 6-(3-(1-(Cyclopentylmethyl)-1H-1,2,3-triazol-4-yl)-6-methylpyridin-2-yl)-2-methylisoindolin-1-one (**16**)

Synthesised according to General Procedure B. The crude product was purified by FCC (DCM → DCM: MeOH 9:1), followed by preparative HPLC (5–100%) to afford the title compound as a yellow oil (37 mg, 33%). ^1^H NMR (CDCl_3_) *δ* 8.07 (d, *J* = 8.0 Hz, 1H), 7.75 (d, *J* = 0.8 Hz, 1H), 7.34 (dd, *J* = 7.8, 1.6 Hz, 1H), 7.25–7.21 (m, 1H), 7.09 (d, *J* = 8.0 Hz, 1H), 6.42 (s, 1H), 4.22 (s, 2H), 3.91 (d, *J* = 7.6 Hz, 2H), 3.03 (s, 3H), 2.44 (s, 3H), 2.08–1.97 (m, 1H), 1.43–1.29 (m, 6H), 0.94–0.85 (m, 2H); ^13^C NMR (CDCl_3_) *δ* 168.1, 157.9, 155.5, 144.7, 141.1, 140.8, 137.3, 133.3, 132.4, 124.5, 122.7, 122.6 (2 ×), 122.2, 54.9, 51.9, 40.6, 30.1, 29.5, 24.9, 24.5; *m/z* MS (TOF ES^+^) 388.2; LC-MS *t*_R_: 2.38; HRMS - C_23_H_26_N_5_O [M+H]^+^ calcd 388.2137; found 388.2141.

#### 4.2.58 3-Azido-2-bromo-6-methylpyridine (**18**)

2-Bromo-6-methylpyridin-3-amine (200 mg, 1.07 mmol. 1.00 equiv.) was dissolved in anhydrous MeCN (5 mL). *tert*-Butylnitrite (127 μL, 1.07 mmol. 1.0 equiv.) was added to the reaction mixture at 0 °C and stirred for 10 min, before the azidotrimethylsilane was added in two potions within 10 min at 0 °C before the reaction mixture was allowed to warm up to room temperature. The reaction was stirred for 1.5 h before it was evaporated to dryness. The residue was purified by FCC (PE 100% → PE: EtOAc 6:4) to afford the title compound as a colourless oil (227 mg, quantitative yield). ^1^H NMR (CDCl_3_) *δ* 7.43 (d, *J* = 8.0 Hz, 1H), 7.24 (d, *J* = 8.1 Hz, 1H), 2.62 (s, 3H); *m/z* MS (TOF ES^+^) no ionization; LC-MS *t*_R_: 2.86.

#### 4.2.59 2-Bromo-3-(4-(cyclopentylmethyl)-1H-1,2,3-triazol-1-yl)-6-methylpyridine (**19**)

Prop-2-yn-1-ylcyclopentane (227 mg, 1.07 mmol, 1.0 equiv.) and 3-azido-2-bromo-6-methylpyridine (127 μL, 1.07 mmol, 1.0 equiv.) were dissolved in mixture of *tert*-BuOH (140 μL, 1.07 mmol, 1.0 equiv.) and water (6 mL, 1:1 ratio). Copper (II) sulfate pentahydrate (53.2 mg, 213 μmol, 0.2 equiv.), followed by sodium ascorbate (84.4 mg, 426 μmol, 0.4 equiv.) were added and the reaction mixture was stirred at room temperature for 17 h. EtOAc (100 mL) was added and the mixture was washed with water (2 × 100 mL) and brine (100 mL). The organic layer was dried with Na_2_SO_4,_ filtered and evaporated to dryness under reduced pressure. The crude product was purified by FCC (PE 100% → PE: EtOAc 4:6) to afford the title compound as a yellow oil (94 mg, 27%). ^1^H NMR (CDCl_3_) *δ* 7.61–7.55 (m, 2H), 7.12 (d, *J* = 8.0 Hz, 1H), 2.63 (d, *J* = 7.4 Hz, 2H), 2.45 (s, 3H), 2.12–2.01 (m, 1H), 1.67–1.58 (m 2H), 1.51–1.32 (m, 4H), 1.13–1.03 (m, 2H); *m/z* MS (TOF ES^+^) 321.0; LC-MS *t*_R_: 3.12.

#### 4.2.60 6-(3-(4-(Cyclopentylmethyl)-1H-1,2,3-triazol-1-yl)-6-

*methylpyridin-2-yl)-2-methylisoindolin-1-one (****20****)* Synthesised according to General Procedure B. The crude product was purified by FCC (DCM → DCM: MeOH 9:1), followed by preparative HPLC (5–100%) to afford the title compound as a yellow oil (27 mg, 23%). ^1^H NMR (CDCl_3_) *δ* 7.99 (d, *J* = 0.9 Hz, 1H), 7.83 (d, *J* = 8.1 Hz, 1H), 7.32 (d, *J* = 8.1 Hz, 1H), 7.26 (d, *J* = 7.8 Hz, 1H), 7.15 (dd, *J* = 7.9, 1.6 Hz, 1H), 6.98 (s, 1H), 4.37–4.30 (m, 2H), 3.18 (s, 3H), 2.69 (s, 3H), 2.61 (d, *J* = 7.4 Hz, 2H), 2.07–1.97 (m, 1H), 1.60–1.41 (m, 6H), 1.08–0.97 (m, 2H); ^13^C NMR (CDCl_3_) *δ* 167.9, 160.1, 152.1, 141.5, 141.4, 137.3, 134.8, 133.6, 131.0, 129.9, 124.2, 123.2, 122.9, 122.6, 51.9, 39.9, 32.2, 31.3, 29.5, 25.0, 24.5; *m/z* MS (TOF ES^+^) 388.2; LC-MS *t*_R_: 2.71; HRMS - C_23_H_26_N_5_O [M+H]^+^ calcd 388.2137; found 388.2136.

### 4.3 Pharmacology

#### 4.3.1 Cell Culture

FlpInCHO cells stably expressing the human M_4_ mAChR (Invitrogen, Carlsbad, CA, USA) were maintained in Dulbecco’s modified eagle’s medium (DMEM) containing 5% FBS and hygromycin B (200 μg/ml) in a humidified atmosphere at 37 °C with 5% CO_2_. Cells were maintained by passaging every 3 days at a 1:10 split ratio, using 0.01% Trypsin, 0.53 mM EDTA diluted in phosphate buffered saline (PBS).

#### 4.3.2 [^3^H]-NMS binding assay

Cells were plated into white Isoplates at 20,000 cells/well and grown overnight at 37 °C. The growth medium was aspirated from the wells, and the cells were washed 1x with PBS, then modified Hank’s Balanced Salt Solution (HBSS: 137 mM NaCl, 5.4 mM KCl, 0.25 mM Na_2_HPO_4_, 0.44 mM KH_2_PO_4_, 4.2 mM NaHCO_3_, 1.8 mM CaCl_2_, 0.8 mM MgSO_4_, 10 mM HEPES, 5 mM D-Glucose, pH 7.4) was added to the wells (70 μL/well). [^3^H]-*N*-Methylscopolamine (NMS) (PerkinElmer Life Sciences) (0.5 nM), ACh (range: 0.01 μM–10 mM) and allosteric modulators (range: 0.1–10 μM) were diluted in HBSS then added to the wells. Plates were incubated on a shaking platform for 6 h at room temperature. Bound [^3^H]-NMS was separated from free [^3^H]-NMS by rapidly aspi-rating the HBSS, then washing the cells 2x with ice-cold NaCl (0.9% w/v). Plates were inverted and dried for 1 h, then UltimaGold scintillant (100 μL/well; PerkinElmer) was added to the plates. Plates were sealed with TopSeal, and each well was counted for 1 min in the microbeta^2^ plate-reader (PerkinElmer).

#### 4.3.3 CAMYEL biosensor activation assay

Cells were plated into white CulturPlates at 20,000 cells/well and grown overnight at 37 °C, then transiently transfected with the CAMYEL biosensor (60 ng/well) using linear polyethyleneimine diluted in 150 mM NaCl (6 μL PEI:1 μg DNA). 48 h later, the growth medium was aspirated from the wells, and the cells were washed 2 × with PBS, then HBSS was added to the wells (70 μL/well). Concentration ranges of Ach and PAMs were diluted in HBSS and mixed together in a drug additions plate. Coelenterazine h (Nanolight technologies) was diluted in HBSS (5 μM). Coelenterazine h, ACh and PAMs were co-added to the wells and incubated at 37 °C for 5 min. Plates were read on the LUMIstar Omega luminometer (BMG LabTech, Offenburg, Germany) using biolumines-cent resonance energy transfer (BRET) 1 filters, with emission filters set at 475 ± 30 nm for detection of eYFP and 535 ± 30 nm for detection of the Rluc8.

#### 4.3.4 β-Arrestin 2 recruitment assay

FlpInCHO cells were plated into white CulturPlates at 20,000 cells/well and grown overnight at 37 °C, then transiently co-transfected with human M_4_-Rluc8 mAChR (20 ng/well) and *β*-arrestin-2-eYFP (80 ng/well) (kind gift from Dr Marc Caron, Duke University) using linear polyethyleneimine diluted in 150 mM NaCl (6 μL PEI:1 μg DNA). 48 h later, the growth medium was aspirated from the wells, and the cells were washed 2 × with PBS, then HBSS was added to the wells (70 μL/well). ACh and PAMs were diluted in HBSS and mixed together in a drug additions plate. Coelenterazine h (Nanolight technologies) was diluted in HBSS (5 μM) and added to the wells for 5 min at 37 °C. ACh and PAMs were then co-added to the wells and incubated at 37 °C for 5 min. Plates were read on the LUMIstar Omega luminometer (BMG LabTech, Offenburg, Germany) with the emission filters set at 475 ± 30 nm for detection of eYFP and 535 ± 30 nm for detection of the Rluc8.

#### 4.3.5 In vivo exposure studies in mice

The study was performed using 10–12 weeks old male C57Bl/6 J naïve mice in accordance with the Australian Code of Practice for the Care and Use of Animals for Scientific Purposes, with procedures approved by the Animal Ethics Committee of the Monash Institute of Pharmaceutical Sciences. The method was the same as previously described [29,30]. **1e, 6k** or **6l** were dissolved in 10% DMSO, 1.1% Tween 80 and 21.8 mM Tris buffer, and administrated in mice by intraperitoneal (i.p.) route at the dose of 10 mg/kg. Mice were euthanised at either 20 or 90 min for all compounds post dosing by cardiac puncture following by cervical dislocation under gaseous anaesthesia (n = 2–3/drug/time point). The concentration of **1e, 6k** and **6l** in the brain parenchyma (C_brain_) were corrected by a subtraction of the compound within the brain vasculature as detailed in our previous studies [32]. Both C_brain_ and compounds concentration in the plasma (C_plasma_) were presented as average concentration (nM) ± SEM in Table 5. The *K*_p_ was then calculated using the formula: K_p_ = C_brain_/C-_plasma_, and presented as ranges.

#### 4.3.6 ERK1/2 phosphorylation assay

The AlphaScreen-based *SureFire* Ultra kit was used for the quantitative measurement of phosphorylated ERK1/2 (pERK1/2). FlpInCHO cells stably expressing the human M_1_-M_5_ mAChRs were used to test compounds for selectivity. 20,000 cells/well were plated into 96 well plates and grown overnight at 37 °C. The next day, the growth medium was aspirated from the wells, and the cells were washed 1 × with PBS, HBSS was added to the wells (80 μL/well), then the cells were incubated for 4 h at 37 °C. ACh (range: 10 nM-10 μM) and PAMs (10 μM) were diluted in HBSS and mixed together in a drug additions plate. ACh and PAMs were co-added to the wells at 37 °C for 5 min. At the end of the stimulation, the HBSS was removed from the wells and 1 × Lysis Buffer (Surefire Ultra kit; 50 μl/well) was added. The plates were frozen at –20 °C overnight, to ensure complete cell lysis. The next day, the lysates were thawed and mixed, and a 5 μL sample was transferred to a white 384 well proxiplate (PerkinElmer). Reaction buffer 1 (1.175 μL/well), reaction buffer 2 (1.175 μl/well), activation buffer (0.1 μl/well) and acceptor beads (0.05 μL/well) were mixed together. Dilution buffer (2.45 μL/well) and donor beads (0.05 μL/well) were mixed together and incubated in the dark for 5 min. Both buffers were mixed together, and 5 μL/well of the combined buffers was added to each well of the proxiplate. Plates were wrapped and incubated at room temperature in the dark for 3–18 h. Plates were read on the Envision multilabel reader (PerkinElmer) using the AlphaScreen settings. Data were expressed as a percentage of the pERK1/2 response to 10% FBS.

#### 4.3.7 Data analysis

Data were plotted and analysed using GraphPad Prism v9.2 (GraphPad Software, SanDiego, CA). All potency, affinity, efficacy and cooperativity values are presented as logarithms [35]. Comparisons between values were made using one-way ANOVA with a Dunnett post-hoc test to determine statistically significant differences compared to the parent compound **1e**, where p < 0.05 was considered significant. The data for the initial screening of the novel allosteric modulators were analysed using the three-parameter logistic equation built into Prism to determine the baseline, potency (pEC_50_) and maximum response (*E*_max_) parameters for the concentration-response curve to ACh with or without the co-addition of the allosteric modulators (1 μM and 10 μM). The Δbaseline, ΔpEC_50_ and Δ*E*_max_ values were calculated by subtracting the value of the ACh curve away from the ACh + PAM curve. The error was propagated using equation (1):

(1) $$Pooled_{SEM}=\sqrt{\;SEM\;1^{2}+SEM2^{2}}$$

Where SEM is the standard error of the mean.

The radioligand binding data were globally fitted with the allosteric ternary complex model according to equation (2) [33]:

(2) $$E=\frac{B_{max\;}\cdot [A]}{[A]+\left(\frac{K_{A}\cdot K_{B}}{\alpha \prime [B]+K_{B}}\right)\left(1+\frac{\left[I\right]}{K_{I}}+\frac{\left[B\right]}{K_{B}}+\frac{\alpha [I][B]}{K_{I}K_{B}}\right)}$$

Where the equilibrium dissociation constants for [^3^H]-NMS, the allosteric ligand and the orthosteric ligand are given by *K*_A_, *K*_B_ and *K*_I_, respectively. [A], [B] and [I] are the concentrations of [^3^H]-NMS, the allosteric ligand and the orthosteric ligand (ACh), respectively. B_max_ is the maximal level of [^3^H]-NMS binding. *α* is the affinity cooperativity between ACh and the allosteric ligand and *α*′ is the affinity cooperativity between the allosteric ligand and [^3^H]-NMS. In some cases, the allosteric ligands have high negative cooperativity with [^3^H]-NMS. Where high negative cooperativity is observed between the allosteric ligand and [^3^H]-NMS, the cooperativity value between the allosteric ligand and [^3^H]-NMS (log*α*′_(NMS)_) is constrained to –3 (*α*′_(NMS)_ = 0.001), to aid model convergence.

For some ligands, the data could not be fitted with the allosteric ternary complex model. For those ligands the data were analysed using the one-site binding equation and the p*K*_I_ was determined using the Cheng and Prusoff correction [36] with equation (3):

(3) $$Y=NSB+\frac{\left(Tot-NSB\right)}{1+\left(\frac{\left[I\right]}{K_{I}\left(1+\frac{\left[A\right]}{K_{A}}\right)}\right)}$$

Where *NSB* is non-specific binding, determined in the presence of 10 μM atropine. *Tot* is total [^3^H]-NMS binding. [*I*] is the concentration of competing ligand, *K*_*I*_ is the equilibrium dissociation constant of the competing ligand in molar. [*A*] is the concentration of [^3^H]-NMS and *K*_*A*_ is the equilibrium dissociation of [^3^H]-NMS.

The p*K*_I_ values were plotted against allosteric modulator concentration and fitted to an allosteric ternary complex model shown in equation (4) [27]:

(4) $$pK_{I}=\log\left(\alpha \times [B]+pK_{B}\right)-logd$$

Where p*K*_I_ is the negative logarithm of the affinity (*K*_I_) value for ACh in the absence or presence of allosteric modulator. p*K*_B_ is the negative logarithm of the dissociation constant of the allosteric modulator determined from radioligand binding assays. *α* is the cooperativity between the allosteric modulator and the orthosteric agonist. d is the estimate of the *K*_I_ in the absence of allosteric modulator. Where the model had difficulty fitting the values, the cooperativity value was estimated using a rectangular hyperbola, where the *E*_max_ was considered to be an estimate for the logα and used to constrain the model to determine an affinity (p*K*_B_) estimate.

CAMYEL activation by four orthosteric agonists was measured. The concentration-response curves were fitted with the operational model of agonism [37] as shown in equation (5):

(5) $$E=\;Basal\;+\frac{\left(E_{max}-\;Basal\;\right)\tau_{A}n{\left[A\right]}^{n}}{\tau_{A}n{\left[A\right]}^{n}+{\left([A]+K_{A}\right)}^{n}}$$

Where Basal is the baseline effect, *E*_max_ is the maximum system response, *τ*_A_ is the operational efficacy of the orthosteric agonist, [A] is the concentration of agonist, *K*_A_ is the affinity of the agonist, fixed to the affinity determined from competition binding between the agonists and [^3^H]-NMS, n is the slope.

The CAMYEL activation data showed changes on the ACh maximal effect (*E*_max_) with increasing concentrations of allosteric modulator indicating that ACh was a partial agonist, therefore full characterisation of the novel M_4_ mAChR PAMs with ACh were fitted to the complete operational model of allosterism and agonism as shown in equation (6) [28]:

(6) $$E=\frac{E_{max}\left(\tau_{A}[A]\left(K_{B}+\alpha \beta [B]\right)+\tau_{B}[B]K_{A}\right)}{\left(\left[A]K_{B}+K_{A}K_{B}+[B]K_{A}+\alpha [A][B\right]\right)+\left(\tau_{A}[A]\left(K_{B}+\alpha \beta [B]\right)+\tau_{B}[B]K_{A}\right)}$$

Where *E*_max_ is the maximum system response, fixed to the *E*_max_ determined from the iperoxo curve (Supp. Fig. 1) fitted with equation (5). [A] is the concentration of ACh and [B] is the concentration of the allosteric modulator. *K*_A_ and *K*_B_ are the equilibrium dissociation constants for ACh and the allosteric modulator, respectively. *τ*_A_ and *τ*_B_ are the operational efficacy for ACh and the allosteric modulator, respectively. α represents the binding cooperativity and *β* is the size of the allosteric effect of the allosteric modulator on the efficacy of ACh. The affinity value (*K*_A_) for ACh was fixed to the affinity determined from competitive equilibrium binding assays, and the affinity value for the allosteric modulators (*K*_B_) were fixed to that determined from binding either using equation (2) or (4), as per above. The transducer slope was fixed to 0.7073 for all analyses.

For the *β*-arrestin recruitment data, no changes in the *E*_max_ of ACh were observed with increasing concentrations of allosteric modulator, therefore the data were fitted to the simplified operational model of agonism and allosterism as shown in equation (7) [38]:

(7) $$E=\;Basal\;+\frac{\left(E_{max}-\;Basal\;\right)([A]\left(K_{B}+\alpha \beta [B]+\tau_{B}[B]EC_{50}\right)}{EC_{50}\left(K_{B}+[B]\right)+([A\left[\left(K_{B}+\alpha \beta [B]\right)+\tau_{B}[B]EC_{50}\right)}$$

Where *E*_max_ is the maximal effect of the system and basal is the baseline response. [A] is the concentration of agonist and [B] is the concentration of allosteric modulator. *K*_B_ is the affinity of the allosteric modulator and EC_50_ is the half maximal response of the orthosteric ligand. *αβ* is the cooperativity between the orthosteric and allosteric ligands and *τ*_B_ is the efficacy of the allosteric ligand. For this analysis the transducer slope (n) was fixed to 1 and the affinity of the allosteric modulator (p*K*_B_) was fixed to the affinity value determined from the radioligand binding data using equation (2) or (4), above.

## Figures and Tables

**Table 1:** Pharmacological evaluation of analogues with modifications to the bottom region, tested at 10 μM against an ACh-mediated concentration-response curve.

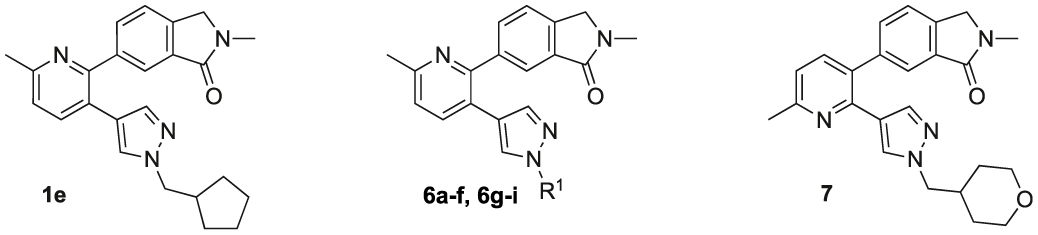				
Compound #	R^1^	Δbaseline^a^	ΔpEC_50_	Δ*E*_max_^b^
**1e**	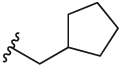	103.2 ± 2.85	1.48 ± 0.20	44.7 ± 2.74
**6a**	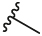	13.7 ± 1.02*	0.57 ± 0.04*	27.0 ± 1.60*
**6b**	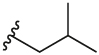	108.5 ± 4.22	1.36 ± 0.20	84.8 ± 5.40*
**6c**	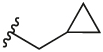	102.5 ± 3.93	1.64 ± 0.20	69.6 ± 4.35*
**6d**	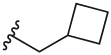	124.1 ± 6.77*	1.05 ± 0.37	74.0 ± 6.52*
**6f**	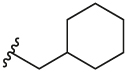	106.4 ± 6.28	0.73 ± 0.39	63.9 ± 7.10*
**6g**	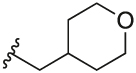	86.7 ± 5.34*	1.16 ± 0.42	47.1 ± 4.88
**6h**	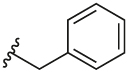	77.1 ± 3.81*	1.87 ± 0.14	63.8 ± 4.05*
**6i**	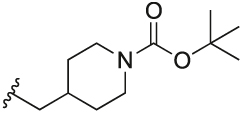	5.53 ± 4.41*	0.60 ± 0.13*	52.5 ± 6.10
**7**		4.76 ± 2.36*	0.58 ± 0.09*	3.58 ± 2.97*

Data represent the mean ± SEM of 4 independent experiments performed in duplicate. Only the change in pEC_50_, baseline and *E*_max_ for the 10 μM concentrations of PAMs are reported, with the full ACh concentration-response curve in the presence of 1 μM and 10 μM PAMs shown in Supp. Fig. 2. Data were analysed by one-way ANOVA and compared to the control PAM, **1e** using a Dunnett post-hoc test

where *p < 0.05 was considered to be significantly different to the control PAM.

a Δbaseline is expressed as a percentage of the maximun ACh response in the absence of PAM.

b Δ*E*_max_ is expressed as a percentage of the maximum ACh response in the absence of PAM.

**Table 2:** Pharmacological evaluation analogues **1e** and **6j**-**u**, tested at 10 μM against an ACh-mediated concentration-response curve.

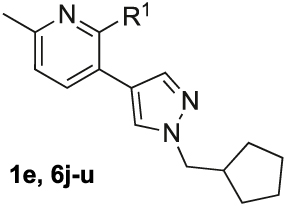				
Compound #	R^1^	Δbaseline^a^	ΔpEC_5__0_	Δ*E*_max_^b^
**1e**	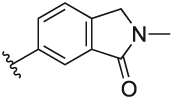	103.2 ± 2.85	1.48 ± 0.20	44.7 ± 2.74
**^6j^**	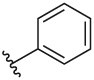	32.1 ± 2.52*	1.15 ± 0.10	21.8 ± 2.97*
**6k**	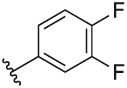	51.4 ± 9.53*	0.76 ± 0.26*	128.8 ± 9.68
**6l**	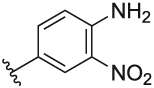	8.48 ± 4.01*	1.27 ± 0.15	5.60 ± 4.45*
**6m**	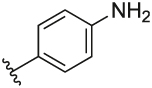	88.1 ± 4.09	1.31 ± 0.17	80.6 ± 4.78*
**6n**	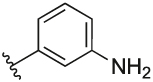	51.7 ± 4.21*	1.35 ± 0.17	53.5 ± 5.54
**6o**	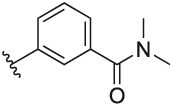	33.2 ± 4.25*	0.25 ± 0.11*	97.7 ± 4.47*
**6p**	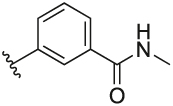	78.2 ± 5.58*	1.99 ± 0.19	61.3 ± 3.81
**6q**	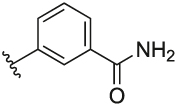	77.3 ± 3.17*	1.33 ± 0.13	65.6 ± 3.48*
**6r**	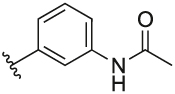	52.2 ± 5.62*	1.63 ± 0.17	57.7 ± 4.85
**6s**	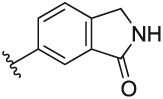	89.7 ± 6.11	2.57 ± 0.25*	73.3 ± 7.00*
**6t**	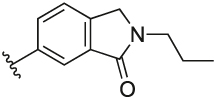	83.3 ± 4.16*	2.11 ± 0.18*	50.5 ± 3.60
**6u**	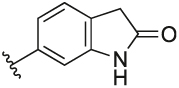	18.6 ± 3.70*	1.24 ± 0.11	39.0 ± 3.53

Data represent the mean ± SEM of 4 independent experiments performed in duplicate. Only the change in pEC_50_, baseline and *E*_max_ for the 10 μM concentrations of PAMs are reported, with the full ACh concentration-response curves in the presence of 1 μM and 10 μM PAMs shown in Supp. Fig. 3. Data were analysed by one-way ANOVA and compared to the control PAM, **1e**, with a post-hoc Dunnett test

where *p < 0.05 was considered to be significantly different.

a Δbaseline is expressed as a percentage of the maximum ACh response in the absence of PAM.

b Δ*E*_max_ is expressed as a percentage of the maximum ACh response in the absence of PAM.

**Table 3:** Pharmacological evaluation analogues **12a-20** tested at 10 μM against an ACh-mediated concentration-response curve.

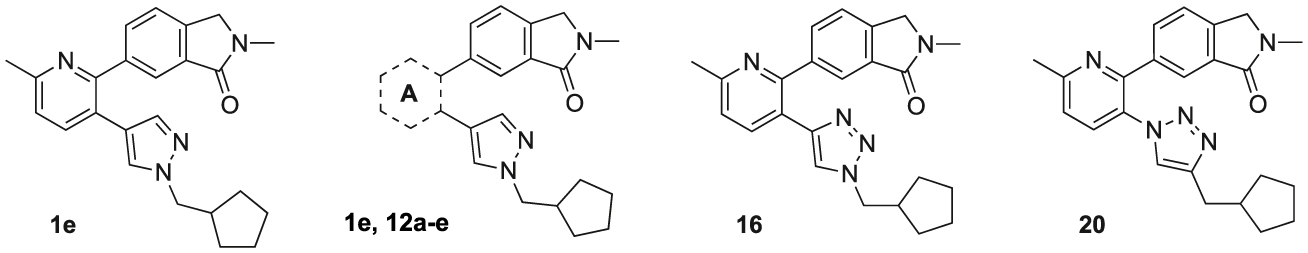				
Compound #	A	Δbaseline^a^	ΔpEC_50_	Δ*E*_max_^b^
**1e**	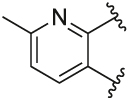	103.2 ± 2.85	1.48 ± 0.20	44.7 ± 2.74
**12a**	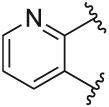	13.0 ± 2.18*	0.88 ± 0.07*	41.6 ± 2.67
**12b**	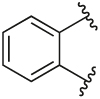	6.49 ± 1.85*	0.63 ± 0.07*	25.6 ± 2.78*
**12c**	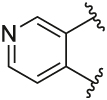	47.2 ± 2.93*	0.17 ± 0.13*	56.8 ± 4.81
**12d**	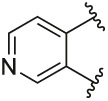	90.6 ± 7.00*	1.49 ± 0.28	66.6 ± 5.99*
**12e**	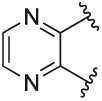	48.5 ± 2.91*	−0.34 ± 0.16*	35.1 ± 5.31
**16**		20.0 ± 4.81*	1.07 ± 0.20	11.8 ± 6.21*
**20**		24.5 ± 2.44*	1.06 ± 0.10	13.9 ± 2.95*

Data represent the mean ± SEM of 4 independent experiments performed in duplicate. Only the change in pEC_50_, baseline and E_max_ for the 10 μM concentrations of PAMs are reported, with the full ACh concentration-response curves in the presence of 10 μM PAMs shown in Supp. Fig. 4. Data were analysed by one-way ANOVA and compared to the control PAM, **1e**, with a post-hoc Dunnett test,

where *p < 0.05 was considered to be significantly different.

a Δbaseline is expressed as a percentage of the baseline ACh response in the absence of PAM.

b Δ*E*_max_ is expressed as a percentage of the maximum ACh response in the absence of PAM.

**Table 4:** Full allosteric quantification of selected analogues.

Comp. #	[^3^H]-NMS binding			CAMYEL activation			*β*-arrestin 2 recruitment	
	p*K*_B_	Log *α*_(ACh)_^a^		Log *τ*_B_^c^	Log *α β*^c^		Log *τ*_B_^d^	Log *α β*^d^
**le**	6.22 ± 0.08^a^	2.12 ± 0.12		− 0.45 ± 0.03	2.34 ± 0.15		0.99 ± 0.10	1.94 ± 0.26
**6c**	5.84 ± 0.04^a^	1.95 ± 0.10		− 0.54 ± 0.03	2.22 ± 0.12		0.64 ± 0.11	1.29 ± 0.25
**6f**	6.11 ± 0.04^a^	1.92 ± 0.10		− 0.88 ± 0.06*	2.01 ± 0.15		0.56 ± 0.06	1.10 ± 0.24
**6j**	4.68 ± 0.11^b^*	1.98 ± 0.13		0.04 ± 0.05*	2.46 ± 0.18		0.87 ± 0.10	1.87 ± 0.18
**6k**	4.53 ± 0.13^b^*	2.04 ± 0.14		− 0.07 ± 0.05*	2.65 ± 0.19		0.31 ± 0.13	1.55 ± 0.16
**6l**	4.75 ± 0.30^a^*	1.87 ± 0.14		− 1.29 ± 0.17*	1.91 ± 0.17		0.39 ± 0.12	1.18 ± 0.23
**6o**	4.49 ± 0.10^b^*	1.47 ± 0.14*		− 0.58 ± 0.09	2.03 ± 0.17		- 0.83 ± 0.53*	1.06 ± 0.19
**12a**	5.33 ± 0.18^a^*	2.18 ± 0.18		− 0.81 ± 0.07*	2.47 ± 0.21		0.51 ± 0.12	2.09 ± 0.15
**12e**	4.55 ± 0.56^b^*	1.41 ± 0.14*		− 0.74 ± 0.09	1.81 ± 0.16		0.07 ± 0.26*	0.94 ± 0.40*

Data are the mean ± SEM of 4 independent experiments with repeats in duplicate. Data were analysed by one-way ANOVA followed by a Dunnett’s post-hoc test

where *p < 0.05 was considered to be significantly different to the parent ligand **1e**.

a Data were fitted with the allosteric ternary complex model (equation (2)), with the affinity of [^3^H]-NMS (Log*K*_A_ = –9.6) and the actual concentration of [^3^H]-NMS (LogHot = –9.3).

b Affinity (p*K*_B_) determined by the global allosteric p*K*_I_ model (Supp. Fig. 6; equation (4)) which was then used to fix the p*K*_B_ value in the allosteric ternary complex model (equation (2)) to derive logα_(ACh)._

c Data fitted with the complete model of allosterism and agonism (equation (6)) where Log*K*_A_ was fixed to the Log*K*_I_ (ACh, –5.17) determined from competition binding between [^3^H]-NMS and ACh. Log*K*_B_ was fixed to the p*K*_B_ determined from the allosteric interaction binding assays. The slope was fixed to 0.7073 which was determined by fitting the iperoxo, ACh, xanomeline and oxotremorine concentration-response curves to a four parameter logistic equation and sharing the slopes. The system *E*_max_ was fixed to 450.9 which was the *E*_*max*_ determined for iperoxo, by fitting concentration-response curves with the operational model of agonism (equation (5)).

d Data were fitted to the simplified operational model of allosterism (equation 7) with Log*K*_B_ fixed to the affinity of the allosteric modulator determined from allosteric interaction binding assays. The slope was fixed to 1.

**Table 5:** Assessment of *in vivo* exposure parameters of **1e** and two novel M_4_ mAChR PAMs.

Cpd	20 or 30 min post-dose		90 min post-dose		
	C_plasma_	C_brain_	C_plasma_	C_brain_	*k*_p_
	nM	nM	nM	nM	
**1e**	2438 ± 858	481 ± 169	14 ± 6	9 ± 5	0.1−0.3
**6k**	915 ± 402	2144 ± 1097	159 ± 64	255 ± 107	1.6−2.5
**6l**	2427 ± 111	982 ± 74	657 ± 35	373 ± 17	0.3−0.6

Data is presented as average ± SEM (n = 2–3/drug/time point), or range for *K*_p_ value.

## Data Availability

Data will be made available on request.

## Abbreviations

ACh: acetylcholine
BRET: bioluminescent resonance energy transfer
Boc: *tert*-butyloxycarbonyl
FlpIn-CHO: Chinese hamster ovary cells with FlpIn vector
cAMP: cyclic adenosine monophosphate
DIPEA: *N,N*-diisopropylethylamine
DMF: *N,N*-dimethylformamide
DCM: dichloromethane
DME: dimethoxyethane
EC_50_: half maximal effective concentration
EtOAc: ethyl acetate
equiv: equivalent
*E*_max_: maximum effect
FCC: flash column chromatography
P-gp: P-glycoprotein
h: hour(s)
HEPES: 4-(2-hydroxyethyl)-1-piperazineethanesulfonic acid
HRMS: high-resolution mass spectrometry
IP: intraperitoneal injection
LCMS: liquid chromatography-mass spectrometry
MeOH: methanol
M_4_: mAChR M_4_ muscarinic acetylcholine receptor
NAM: negative allosteric modulator
NMP: *N*-methyl-2-pyrrolidone
NMR: nuclear magnetic resonance
SEM: standard error of the mean
PAM: positive allosteric modulator
PE: petroleum spirits 40-60
PET: positron emission tomography
*t*-BuOH: tertiary butanol
TFA: trifluoroacetic acid
THF: tetrahydrofuran
TMS: trimethylsilyl
TLC: thin layer chromatography
rt: room temperature
